# Janus kinase inhibitors in atopic dermatitis: an umbrella review of meta-analyses

**DOI:** 10.3389/fimmu.2024.1342810

**Published:** 2024-02-23

**Authors:** Qingying He, Xin Xie, Qian Chen, Wenquan Li, Zongzhou Song, Xurui Wang, Xiao Ma, Jinhao Zeng, Jing Guo

**Affiliations:** ^1^Dermatological Department, Hospital of Chengdu University of Traditional Chinese Medicine, Chengdu, China; ^2^School of Clinical Medicine, Chengdu University of Traditional Chinese Medicine, Chengdu, China; ^3^State Key Laboratory of Southwestern Chinese Medicine Resources, School of Pharmacy, Chengdu University of Traditional Chinese Medicine, Chengdu, China; ^4^TCM Regulating Metabolic Diseases Key Laboratory of Sichuan Province, Hospital of Chengdu University of Traditional Chinese Medicine, Chengdu, China

**Keywords:** Janus kinase inhibitors, atopic dermatitis, inflammatory network, umbrella review, meta-analyses

## Abstract

**Background:**

Clinicians and healthcare policymakers have been drenched with a deluge of overlapping meta-analyses (MAs), and the necessity for comprehensive and clearly defined evidence of Janus kinase inhibitors (JKIs) in atopic dermatitis (AD) is urgent.

**Methods:**

Six databases were searched for MAs published until October 2023. Qualitative description of MAs was mainly used, and Investigator's Global Assessment response (IGA response), the 75% improvement in Eczema Area and Severity Index (the EASI75), peak pruritus Numerical rating score (PP-NRS), and adverse effects were cited to describe the efficacy and safety of JKIs. The methodological quality of the included MAs was assessed by A Measurement Tool to Assess Systematic Reviews II (AMSTAR II), and the quality of evidence was evaluated by the grading of recommendations, assessment, development, and evaluation (GRADE).

**Results:**

Sixteen MAs were pooled in this review, of which five studies appraised JKIs, five appraised systemic JKIs, five papers assessed abrocitinib only, and one assessed baricitinib. Two studies were of “high” methodological quality and 14 MAs were of “moderate” quality. Eleven MAs integrated the results of JKIs and reported that JKIs provide faster onset of IGA response (RR=2.83, 95% CI [2.25, 3.56], high-quality evidence). Similarly, 10 MAs showed that JAK inhibitors were more effective in improving the EASI75 (RR=2.84, 95% CI [2.2, 3.67], high-quality evidence). Results from 12 MAs showed JKIs were active in reducing the PP-NRS (SMD=-0.49, 95% CI [-0.67, -0.32]). All MAs affirmed JKIs added no adverse effects leading to discontinuation and serious adverse events (P<0.05). However, 200mg of abrocitinib had a higher risk of acne (RR=4.34, 95% CI [1.61, 11.71), herpes zoster (RR=1.64, 95% CI [0.42, 6.39]), headache (RR=1.76, 95% CI [1.03, 3]), and nausea (RR=7.81, 95% CI [3.84, 15.87]). Upadacitinib was known to increase acne (RR=6.23, 95% CI [4.08, 9.49]), nasopharyngitis (RR=1.36, 95% CI [1.03, 1.8]) and blood creatine phosphokinase (blood CPK) (RR=2.41, 95% CI [1.47, 3.95]). Baricitinib at 2mg was associated with increased blood CPK (RR=2.25, 95% CI [1.1, 2.97]).

**Conclusion:**

Compared to placebo or dupilumab, the administration of JKIs can ameliorate IGA response more effectively, improve the EASI75, and relieve pruritus without severe adverse effect, while accompanied by more acne, nasopharyngitis, headache, and digestive disturbances. The curative effect of 200 mg of abrocitinib is significant and more caution should be given in patients with gastrointestinal dysfunction, herpes zoster, and those who are acne-prone. Baricitinib and upadacitinib should be avoided in populations at high risk for cardiovascular events.

**Systematic review registration:**

https://www.crd.york.ac.uk/prospero/display_record.php?RecordID=369369, PROSPERO (CRD42022369369).

## 1 Introduction

Atopic dermatitis (AD) is one of the most common inflammatory skin conditions, which is characterized by widespread eczematous lesions with severe pruritus and an increased risk of skin infection (1). Generally, AD occurs in childhood and is highly likely to persist into adulthood. The prevalence of AD ranges from 15~20% in children to about 10% in adults (2). The high recurrence rate and long-term treatment of AD not only severely reduce the quality of life of patients but also make them susceptible to low self-esteem, anxiety, depression, and even suicidal impulses (3). These have a negative impact on patients’ quality of life and psychology while bringing about an increase in overall healthcare costs; a report shows that 22% of patients spend more than $12,000 per year to treat atopic dermatitis and 68.7% of patients visit the clinic an average of one to three times in a month (4).

The pathogenesis of AD is not fully clarified, and is intimately related to genetic, environmental, and immune abnormalities and skin flora disorders. However, there is no doubt that AD is the result of an integrated disturbance of the inflammatory network. Th2-type inflammation is the basic feature of AD, and IL-4, IL-13, and IL-31 are important cytokines mediating the development of AD (5–7). Current domestic and international guidelines recommend a series of treatments for AD patients according to disease severity; mild patients can be treated with topical glucocorticoids (TCS) and topical calcium phosphatase inhibitors (TCI), of which TCS is the first-line therapy. Patients with moderate pruritus require combined topical drugs and active maintenance therapy with TCI/TCS; furthermore, oral antihistamines and antimicrobials or combined phototherapy are recommended if necessary. Systemic immunosuppressive agents, biologics, and phototherapy are recommended for patients with severe pruritus (8). Current research indicates that these treatments are prone to adverse effects such as skin atrophy and thinning, hyperpigmentation, secondary infections, and drug dependence (9–11).

**Figure 1:**
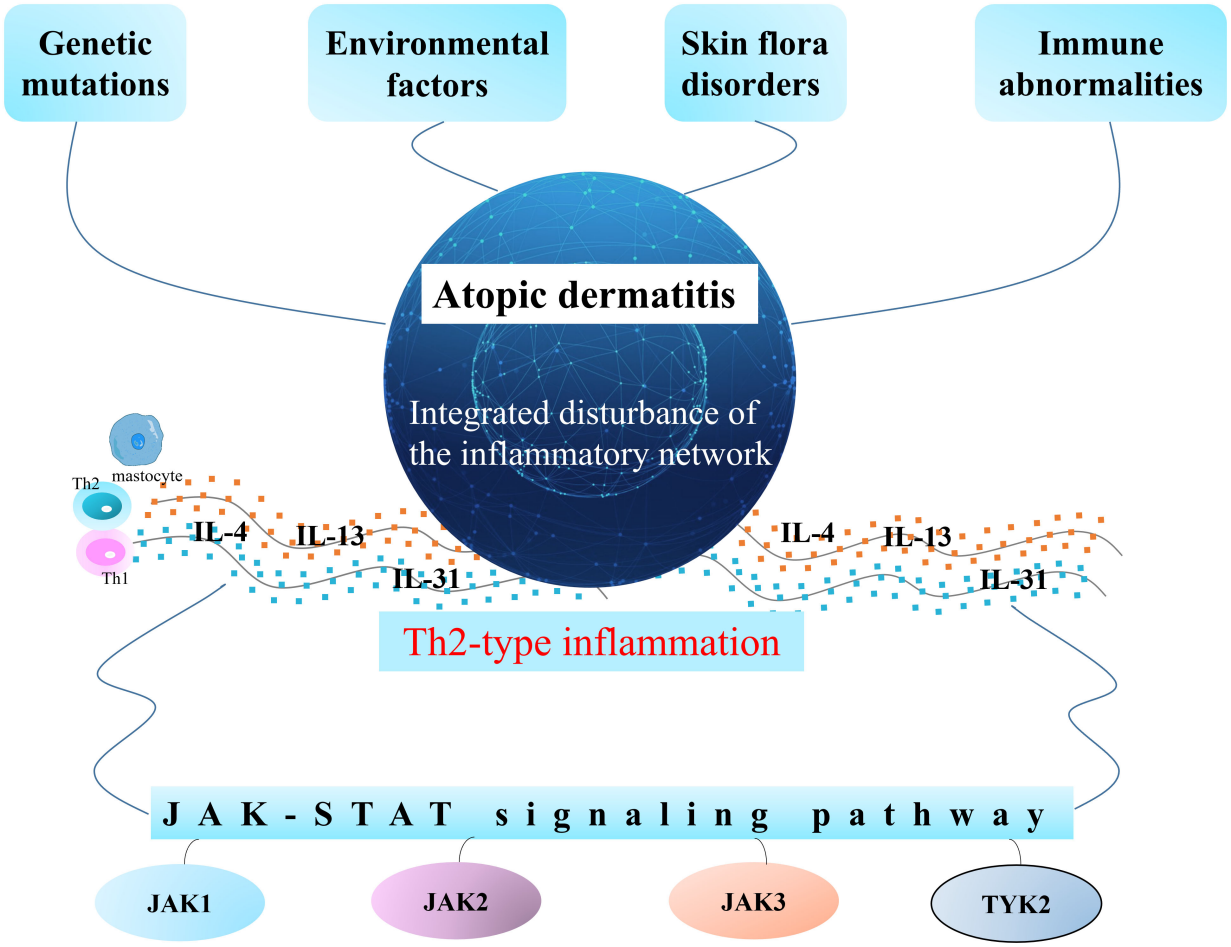
Mechanism of atopic dermatitis and current status of JKIs management.

Complex diseases are associated with multiple mechanistic pathways, among which research around Janus kinase (JAK) has attracted much attention due to their broad regulation of AD, and many new small molecule targeted drugs and biologics have been developed (12–14). Janus kinase inhibitors (JKIs) are recommended by guidelines to block a variety of signaling factors involved in the immune response and inflammation (15). To date, eight drugs have been successfully approved for marketing worldwide, and dozens of new drugs are still in clinical development. The abbreviated mechanism of atopic dermatitis is shown in **Figure 1**.

Despite the fact that several meta-analyses (MAs) on JKIs for AD were published in October 2022 (16–19), a great number of related MAs have been published in just one year (20–23). It is apparent that the usage of JKIs in AD is still widely discussed. There is no doubt that these MAs provide guidance to clinicians on the application of JKIs; however, it is undeniable that many of these MAs are duplicative and their methodological quality has not been assessed, resulting in a continued lack of confidence in JKIs among clinical practitioners and patients. Currently, an umbrella review of meta-analyses is considered a comprehensive evidence-summarizing research strategy that can build on MAs to further summarize research information in the field and reduce uncertainty in decision making (24). Hence, this umbrella review aims to provide an umbrella to prevent clinical practitioners and healthcare policymakers from being drenched by a deluge of evidence, as well as to keep the wider scope of evidence within a specific context.

## 2 Methods

We conducted this umbrella review in accordance with the Joanna Briggs Institute Umbrella Review Methodology (25). The study protocol was registered on PROSPERO (registration number: CRD42022369369, URL: https://www.crd.york.ac.uk/prospero/display_record.php?RecordID=369369).

### 2.1 Search strategy

After determining the search formula based on the study objectives, two researchers conducted a comprehensive search on four English databases (Pubmed, Embase, Web of Science, and Cochrane Library) and two Chinese databases (China National Knowledge Infrastructure and Wanfang Data Knowledge Service Platform). The search was limited to the period from the establishment of each database to October 20, 2023. The search strategy for each database is shown in **Supplementary Table 1**. Google Scholar, Baidu Scholar, and the references of relevant reviews were reviewed in case of omission. We only included Chinese and English literature, but placed no restriction on publication country or publication status (including gray literature).

### 2.2 Inclusion exclusion criteria

Our aim was to identify all MAs which summarized and reported the efficacy and safety of JKIs in atopic dermatitis. Inclusion and exclusion criteria were based on Population, Intervention, Control, Outcome and Study type (PICOS) elements.

#### 2.3.1 Inclusion criteria

Patients: Patients diagnosed with AD (the patients were determined by a dermatologist referring to atopic dermatitis diagnosis guidelines, such as the “European guideline on atopic eczema” or “Chinese Atopic Dermatitis Diagnosis and Treatment Guidelines”) (8, 26), regardless of age, gender, race, ethnicity, and disease duration.

Intervention and control group: JKIs (either topical or systemic) were used in the intervention group and were compared with placebos or conventional medication.

Outcomes: Studies reporting on any of the following indicators were included: ① the percentage of patients who achieved an Investigator’s Global Assessment score (IGA) of 0 or 1; ② the percentage of patients with 75% improvement in the Eczema Area and Severity Index of 75% (EASI75); ③ the Peak Pruritus Numerical Rating Scale (PP-NRS); or ④ relative safety indicators.

Study type: Systematic review with meta-analyses of randomized controlled trials.

#### 2.3.2 Exclusion criteria

(1) Study subjects with AD combined with other skin diseases (contact dermatitis, pemphigus, etc.) (2); MAs of part of the original studies included where the intervention group was non-JAK inhibitors (3); the use of evaluation indicators that were non-efficacy and safety indicators, such as economic evaluation and mechanism of action reviews (4); systematic reviews without meta-analyses, network meta-analyses, protocols, pooled analyses, narrative review, guidelines, expert consensus, or studies based on non-humans (such as animals) (5); conference papers, letters to the editor, and so on (6); literature published in languages other than Chinese or English; and (7) studies for which the full text was not available.

### 2.3 Study selection and data extraction

Two researchers imported the retrieved literature into Endnote (version 9.1) to remove duplicates and then screened the literature back-to-back based on the inclusion criteria. The screening process was as follows. Firstly, we read the title and abstract of the article to determine the “initial inclusion pool” based on whether the population, intervention, and study type met the inclusion criteria. Then, we downloaded the full text and excluded non-compliant studies based on the exclusion criteria to finalize the studies for inclusion in this review.

The research team developed a literature extraction form, and two researchers independently extracted the data for inclusion in the study. The following information was extracted (1): basic information such as title of the article, year of publication, name of the first author, affiliation, study design, number of original studies included, total sample size, and period of the search (2); quality assessment tools used and quality assessment results, conflict of interest, publication bias, and funding source; and (3) quantitative analysis methods (random or fixed effects) and main results (outcome indicators, estimates of effect or association and their *P* value or 95% confidence interval, and heterogeneity).

### 2.4 Assessment of methodological quality

A Measurement Tool to Assess Systematic Reviews II (AMSTAR II) was used to assess the methodological quality of the included MAs and contained 16 questions. For the convenience of summarizing statistics, we made a small adjustment to the entry order and result determination of the AMSTAR II, as follows (1): Did research questions and inclusion criteria include PICO? (2) Were the study methods reported to have been established prior to implementation and the inconsistencies with the protocol described? (3) Were the types of studies included and the reasons for their selection described? (4) Was a comprehensive search strategy used? (5) Was the literature screening process replicable? (6) Was the data extraction process replicable? (7) Were the appropriate tools used to assess the risk of bias of the included studies? (8) If meta-analysis was performed, were appropriate statistical methods used to combine results? (9) Was an exclusion list provided after reading the full text, with reasons for exclusion? (10) Was the underlying information such as the PICOS of the included studies described in detail? (11) Was the funding information reported for each of the included studies? (12) If meta-analysis was performed, was the effect of bias on the results of individual studies considered? (13) Did the systematic evaluation authors consider the risk of bias when interpreting or discussing the study results? (14) Did the authors of the systematic evaluation interpret or discuss the heterogeneity of the results? (15) If a meta-analysis was conducted, was publication bias investigated and its impact on the results discussed? (16) Did the authors of the systematic evaluation report any potential conflicts of interest, including the financial support received to conduct the systematic review (SR)? Items 2~10, 15, and 16 were answered “yes”, “no”, or “partly yes”, and items 1 and 11~14 were answered “yes” or “no”.

Two investigators conducted the methodological assessment of the included MAs independently and discussed with senior investigators in the team to reach a consensus after meeting differences. To quantify the methodological quality assessment, we assigned a score of 2 to “yes”, 1 to “partially yes”, and 0 to “no”, with a total score of 32. Studies with a score ≥25 indicated that we were unanimous about their methodological quality with high confidence; a score of 17~24 indicated moderate confidence, 9~16 was low confidence, and ≤8 was particularly low.

### 2.5 The credibility of evidence

The grading of recommendations, assessment, development, and evaluation (GRADE) was used to assess the quality of the available evidence for the primary outcome (27), taking into account five downgrading factors (study limitations, inconsistency, indirectness, imprecision, and publication bias) and three upgrading factors (large effect size, reasonable confounding to increase the confidence in the estimated effect, and dose-response relationship). The specific interpretation criteria for each escalation factor are shown in **Supplementary Table 2**. The quality of evidence was classified as “high”, “moderate”, “low”, and “very low” based on the results.

### 2.6 Synthetic analysis of MAs

Qualitative description of research results was mainly used, based on the follow information for each study (1): the number of included RCTs (2), the effect size used (Relative risk, RR. Odds ratio, OR. Standardized mean difference, SMD), and (3) the estimate of effect, along with their corresponding 95% confidence interval (95% CI). In addition, we integrated all meta-analyses that reported the same outcome indicator and compared them visually by different disease severity, age, and dosage. All data were analyzed by GraphPad Prism 8 and Stata 16.

## 3 Results

### 3.1 Literature screening results

**Figure 2:**
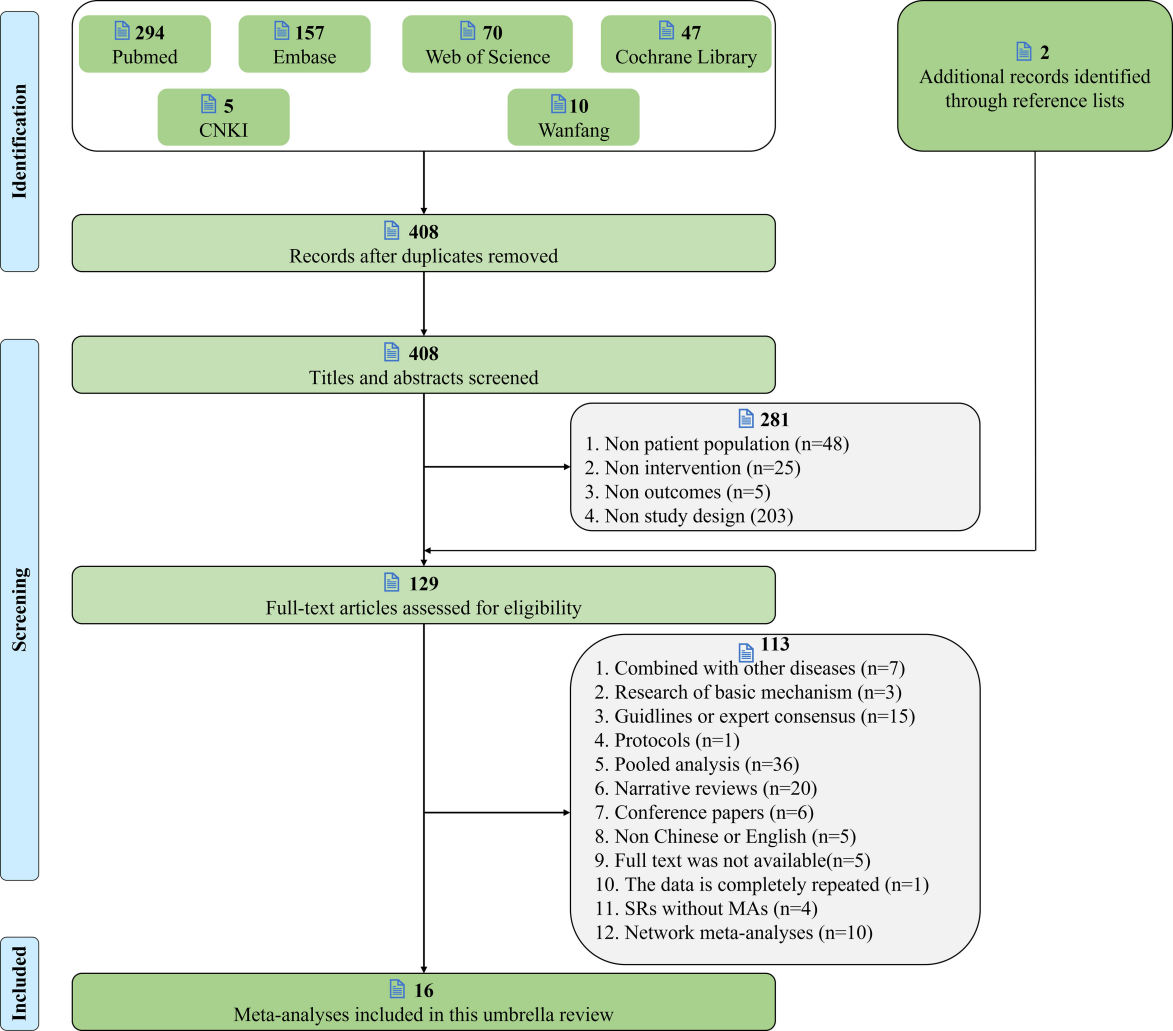
Literature screening flow chart.

A total of 583 records were obtained through six electronic databases. After removing duplicate items, the titles and abstracts of 408 documents were screened, 127 potentially relevant studies were identified, 2 potentially eligible articles were obtained through additional searches, and 16 meta-analyses were finally included after downloading the 129 full text reviews. The flow chart of literature screening is shown in **Figure 2**.

### 3.2 Basic characteristics of the included studies

The characteristics of the included MAs are presented in **Table 1**. These MAs enrolled randomized controlled studies conducted by October 2023. The papers were published from July 2020 to September 2023, accounting for six in 2023, seven in 2022, two articles in 2021, and the remaining one in 2020. By language of publication, 15 were in English and one was in Chinese; as for the affiliation of the first author, 11 were from China (of which nine were from mainland China and two from Taiwan), and one each was from India, Saudi Arabia, Korea, the United States, and Australia.

**Table 1:** Characteristics of included meta-analyses.

Author (Year)	Affiliation	Search duration	Studies included, sample size	Age of participants (years)	AD severity	Intervention	Control	Treatment duration (weeks)	Efficacy outcome	Safety outcome	Subgroup analysis
Chen JS (2022) (28)	China	Inception-2021.9	22 articles (25 RCTs) N=9,931	No limitation	NR	JKIs	Placebo or vehicle	NR	*Change in EASI *Change in PP-NRS *IGA response	*TEAEs * AEs leading to drug discontinuation *Patients with serious AEs	*Administration routes
Tsai HR (2021) (29)	China Taiwan	Inception-2021.2	14 articles (15 RCTs) N=4,367	No limitation	No limitation	JKIs	Placebo or vehicle	4, 8, 12, 16	*EASI75 *IGA response *PP-NRS response	*TEAEs *AEs leading to drug discontinuation	*Administration route *Treatment duration *Baseline severity *Age of participants *Mechanism of action
Miao MY (2022) (30)	China	Inception- 2021.1	9 articles (10 RCTs) N=2,583	No limitation	NR	JKIs	Placebo or vehicle	4, 12, 16, 24	*Change in EASI *Change in PP-NRS *EASI50 *EASI75 *EASI90	*TEAEs	*Day-time or Night-time for NRS
Li CY (2022) (17)	China	Inception- 2020.9	13 articles (14 RCTs) N=3,822	No limitation	No limitation	JKIs	Placebo or vehicle	4, 8, 12, 16	*Change in EASI *IGA response	*TEAEs *AEs leading to drug discontinuation	*Administration route *Type of JKIs *Treatment duration *Baseline severity
Arora CJ (2020) (31)	Australia	Inception-2019.9	5 articles (5 RCTs) N=658	No limitation	No limitation	JKIs	Placebo or vehicle	4, 16	*Change in EASI *Change in PP-NRS	/	No
Sun C (2023) (21)	China	2020.1-2022.10	10 articles (10 RCTs) N=7,901	No limitation	No limitation	Systemic JKIs	Placebo or dupilumab	12, 16, 24, 26, 40	/	*Acne	*Drug dosages
Sanghyuk (2023) (22)	Korea	2019-2022.6	14 articles (16 RCTs) N=7,543	No limitation	No limitation	Systemic JKIs	Placebo	16, 40, 52	/	*TEAEs *Serious adverse events *Severe adverse events	*Type of JKIs *Drug dosages
Chen TL (2022) (32)	China Taiwan	Inception-2022.2	15 articles (17 RCTs) N=8,545	No limitation	No limitation	Systemic JKIs	Placebo or dupilumab	12, 16, 24, 40	/	*Venous thromboembolism	*Type of JKIs
Kevin P (2023) (33)	USA	Inception-2021.9	12 articles (14 RCTs) N=6,653	≥12	No limitation	Systemic JKIs	Placebo	12, 16	*Change in EASI *EASI75 *IGA response * PP-NRS response	*TEAEs	*Type of JKIs *Drug dosages
Gao Q (2023) (34)	China	1950-2022.9	3 articles (3 RCTs) N=2,256	>18	Moderate-to-severe	Abrocitinib, upadacitinib	Dupilumab	16, 24, 26	*EASI75 *EASI90 *IGA response * PP-NRS response	*TEAEs *Serious adverse events of any cause *Severe adverse events of any cause	*Treatment duration
Li L (2023) (35)	China	Inception-2022.8	7 articles (6 RCTs) N=3,440	≥12	Moderate-to-severe	Abrocitinib	Placebo or dupilumab	12, 16, 26	*EASI75 *IGA response * PP-NRS response *PSAAD *POEM *DLQI *HADS	*TEAEs *Serious adverse events of any cause	*Drug dosages *Treatment duration *Age of participants
Liu SQ (2023) (36)	China	Inception-2022.6	5 articles (5 RCTs) N=1,825	≥12	Moderate-to-severe	Abrocitinib	Placebo	12, 16	*EASI75 *IGA response * PP-NRS response	*TEAEs *Serious adverse events of any cause	*Drug dosages
Zhang DJ (2022) (37)	China	Inception- 2021.6	4 articles (4 RCTs) N=590	No limitation	No limitation	Abrocitinib	Placebo	NR	*EASI75 *EASI90 *IGA response * PP-NRS response	*TEAEs *Serious adverse events of any cause	No
Bikash (2022) (38)	India	Inception- 2021.4	4 articles (4 RCTs) N=1,843	≥12	Moderate-to-severe	Abrocitinib	Placebo	12, 16	*IGA response *EASI75 * PP-NRS response	*TEAEs	*Drug dosages
Hammad (2021) (39)	Saudi Arabia	Inception-2021.2	4 articles (4 RCTs) N=1,882	No limitation	Moderate-to-severe	Abrocitinib	Placebo	12, 20	*EASI75 *EASI90 *IGA response * PP-NRS response *PSAAD *POEM *DLQI	*TEAEs *Serious adverse events of any cause	*Drug dosages
Wang B (2022) (19)	China	Inception- 2021.7	5 articles (6 RCTs) N=2,595	>18	moderate-to-severe	Baricitinib	Placebo	16	*EASI75 *EASI90 *IGA response * PP-NRS response *SCORAD75	*TEAEs	* Drug dosages

JKIs, Janus kinase inhibitors; NR, not reported; EASI, Eczema Area and Severity Index; IGA, Investigator Global Assessment; PP-NRS, Peak Pruritus Numerical Rating Scale; POEM, Patient Oriented Eczema Measure; DLQI, Dermatology Life Quality Index; PSAAD, Pruritus and Symptoms Assessment for Atopic Dermatitis; HADS, Hospital Anxiety and Depression Scale; SCORAD, Scoring Atopic Dermatitis; TEAEs, treatment-emergent adverse events; AEs, adverse events.

Regarding population characteristics, two MAs included only RCTs conducted on adults (>18 years old), four studies included people ≥12 years old, and 10 studies specified no limitation on age. Six MAs specifically stated that they only included patients with moderate to severe atopic dermatitis, and the remaining studies did not specifically report the severity of atopic dermatitis.

Of the 16 MAs included, five studies appraised JKIs (control group were placebo or vehicle), and the count of original studies included ranged from five to 25 RCTs, with a maximum sample of 9,931 participants. Five assessed systemic JKIs (two versus placebo, one versus dupilumab, and two versus dupilumab or placebo); the most representative MAs enrolled 8,545 individuals with AD from 17 RCTs. Five papers assessed abrocitinib only (four versus placebo and one versus dupilumab or placebo) and the remaining one assessed baricitinib (versus placebo). The majority of the studies reported follow-up periods, which ranged from 4 weeks to 52 weeks.

Among these MAs, three papers were devoted to reporting the safety of systemic JKIs, 12 focused on both efficacy and safety outcomes, and the remaining one elicited only the efficacy of JKIs. Fourteen MAs performed subgroup analyses with route of administration, drug dosage, or type of JKIs.

### 3.3 Methodological quality of the included studies

**Figure 3:**
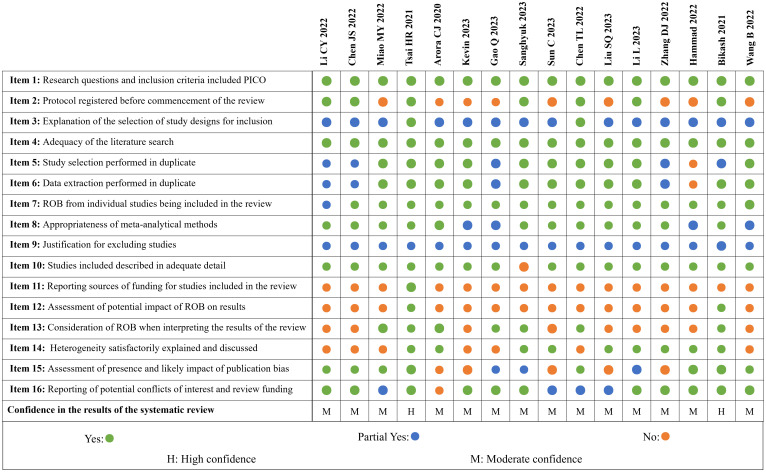
Quality assessment of included reviews based on AMSTAR-2.

**Figure 3** shows the overall results of the AMSTAR-II. We have “high confidence” in the methodological quality of two MAs and 14 studies received “moderate confidence”.

All studies reported PICO elements in the inclusion criteria and used ≥2 electronic databases; seven studies underwent registration of research protocols and received two points each. Two studies (29, 32) described the types of studies included and gave reasons for their selection, while 14 studies only described the types of studies included. Ten studies used a two-person back-to-back approach for screening and extraction and described the solution when disagreement was encountered; five MAs (17, 28, 34, 37, 38) were lacking a cross-review process and one study (39) did not describe this process at all. One study (17) did not score on item 7 due to not assessing the methodological bias for inclusion in the RCT. Four studies (19, 33, 34, 39) did not state how to deal with heterogeneity and received only one point each in item 8. All MAs stated the reasons for exclusion but did not provide a list of exclusions. All but one study (22) described the baseline characteristics of the included RCTs in detail. Only one study (29) described the source of funding for the original RCTs, and two studies (29, 38) considered the impact of the risk of bias of the included studies on meta analyses; however, seven studies considered the risk of bias in the discussion of the MAs. Additionally, nine studies considered the heterogeneity of results in the discussion and eight studies fully investigated publication bias and discussed its impact on outcomes. Four studies (22, 30, 32, 36) did not describe any conflict of interest, and one (31) did not report either the funding grant or the declaration of interest.

### 3.4 Summary of evidence on the efficacy and safety of JKIs

#### 3.4.1 The efficacy of JKI inhibitors

##### 3.4.1.1 IGA response

**Figure 4:**
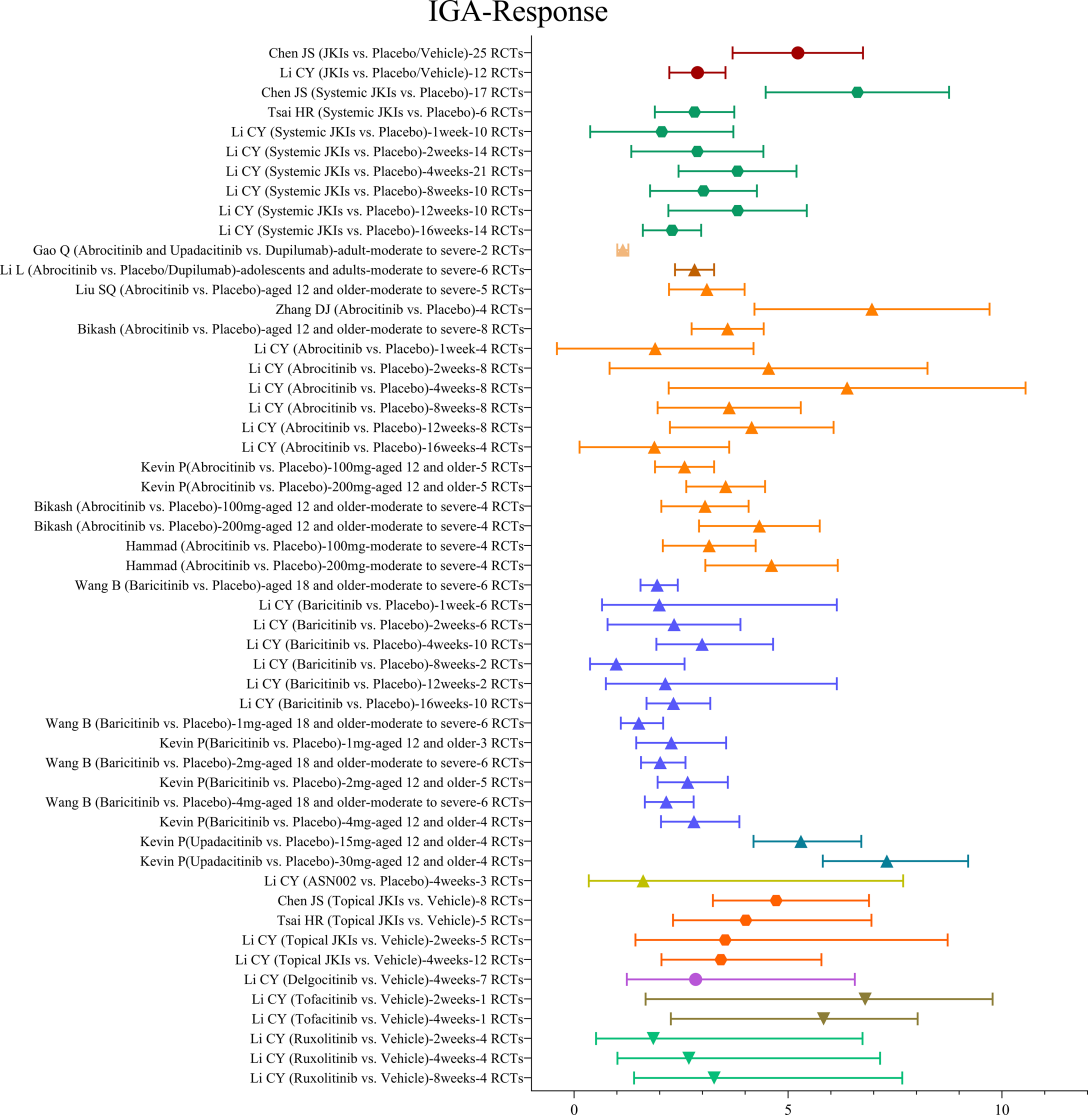
The summary evidence of IGA response.

Eleven MAs (17, 19, 28, 29, 33–39) integrated the results of JKIs on IGA response; the summary evidence is shown in **Figure 4**. Overall, two MAs (17, 28) with 25 RCTs reported that, compared with placebo or vehicle, JKIs provide faster onset of IGA response (RR=2.83, 95% CI [2.25, 3.56]).

Three studies (17, 28, 29) demonstrated the effectiveness of systemic JKIs. Similarly, systemic JKIs were found to improve IGA response more effectively (RR=2.71, 95%CI [1.94, 3.79]) compared with placebos. Li CY (17) evaluated the impact of various treatment courses and discovered that systemic JKIs exhibited greater efficacy beyond one-week courses; nevertheless, no significant dose-response gradient was observed.

All six studies proved that (17, 33, 36–39), compared to placebo, abrocitinib is favorable in IGA response in moderate to severe atopic dermatitis in adolescents and adults (RR=3.02, 95%CI [2.26, 4.02]). From the various intervention durations, the advantage of abrocitinib was not statistically significant at either one week (RR=0.96, 95% CI [0.21, 4.51]) or 16 weeks (RR=1.33, 95% CI [0.46, 3.83]) (17). The most notable effect on IGA was observed at week 4 (RR=5.47, 95% CI [2.74, 10.93]); however, it is important to note that these results were obtained from a single study (17). Pooled data from five RCTs showed that 200 mg of abrocitinib was superior to 100 mg in improving IGA response (RR=2.52, 95% CI [1.92, 3.3]) (33), and two additional MAs (38, 39) confirmed the findings.

Wang B (19) appraised the results of six RCTs of baricitinib in adult patients with moderate-to-severe atopic dermatitis, which were able to reduce IGA scores more promptly (RR=1.94, 95% CI [1.55, 2.42]) as compared with placebos. Subgroup analyses of the intervention course showed that baricitinib did not reveal greater benefits than placebos at the first, second, eighth, and twelfth week (P>0.05); furthermore, the most marked result on IGA response was at the fourth week (RR=2.99, 95% [1.92, 4.65]) (17). Two MAs (19, 33) compared the efficacy of doses and showed that higher doses of baricitinib increased the efficiency figure of IGA response, but the differences were not statistically significant (P>0.05).

One MA (33) which included four RCTs affirmed that upadacitinib stabilized IGA response rapidly (RR=5.3, 95% CI [4.19, 6.71]). One MA (17) with three RCTs showed that gusacitinib has no ameliorative effect on IGAs response (RR=1.61, 95% CI [0.34, 7.69]) compared with those treated with placebos.

Only one study (34) performed the JKIs against dupilumab and showed a slight advantage in IGA response for abrocitinib or upadacitinib (RR=1.13, 95% CI [1.01, 1.27]).

Three studies (17, 28, 29) have summarized the efficacy of topical JKIs on IGA response, and the most recent evidence demonstrated that, as compared with placebos, topical JKIs decreased IGA scores for a greater proportion of patients (RR=3.43, 95% CI [2.04, 5.78]).

##### 3.4.1.2 EASI75

Similarly, 10 MAs pooled the results of the EASI75. One MA (29) reviewed 12 RCTs with 4,499 participants and showed that, in comparison to placebos, JKIs were more effective in improving the EASI75 without significant heterogeneity (RR=2.84, 95% CI [2.2, 3.67]). Subgroups of participants were analyzed by age and severity, and it was surprising to detect that compared adults only (RR=2.21, 95% CI [1.78, 2.74]), studies with children and adolescents obtained higher EASI75 responses (RR=4.68, 95% CI [3.23, 6.79]).

Six MAs (33, 35–39) assessed the EASI75. All showed that abrocitinib could achieve improvements in the EASI75 more efficiently than placebos. Several studies have showed that while the improvement in the EASI75 with 200 mg (RR=3.04, 95% CI [2.22, 4.16]) of abrocitinib was numerically higher than 100 mg (RR=2.18, 95% CI [1.78, 2.67]), there was no statistical difference between the dosages (35, 36, 39).

Two MAs (19, 33) were conducted to assess the effect of baricitinib on the EASI75. The results yielded a greater improvement in efficacy with 2mg (RR=2.46, 95% CI [1.89, 3.18]) compared to 1mg (RR=1.87, 95% CI [1.3, 2.69]) but no significant improvement with 4mg (RR=2.57, 95% CI [1.95, 3.38]) compared to 2mg, irrespective of disease severity. Either 15mg (RR=3.48, 95% CI [3.01, 4.03) or 30mg (RR=4.14, 95% CI [3.59, 4.77) of upadacitinib was very effective in the EASI75 (33).

**Figure 5:**
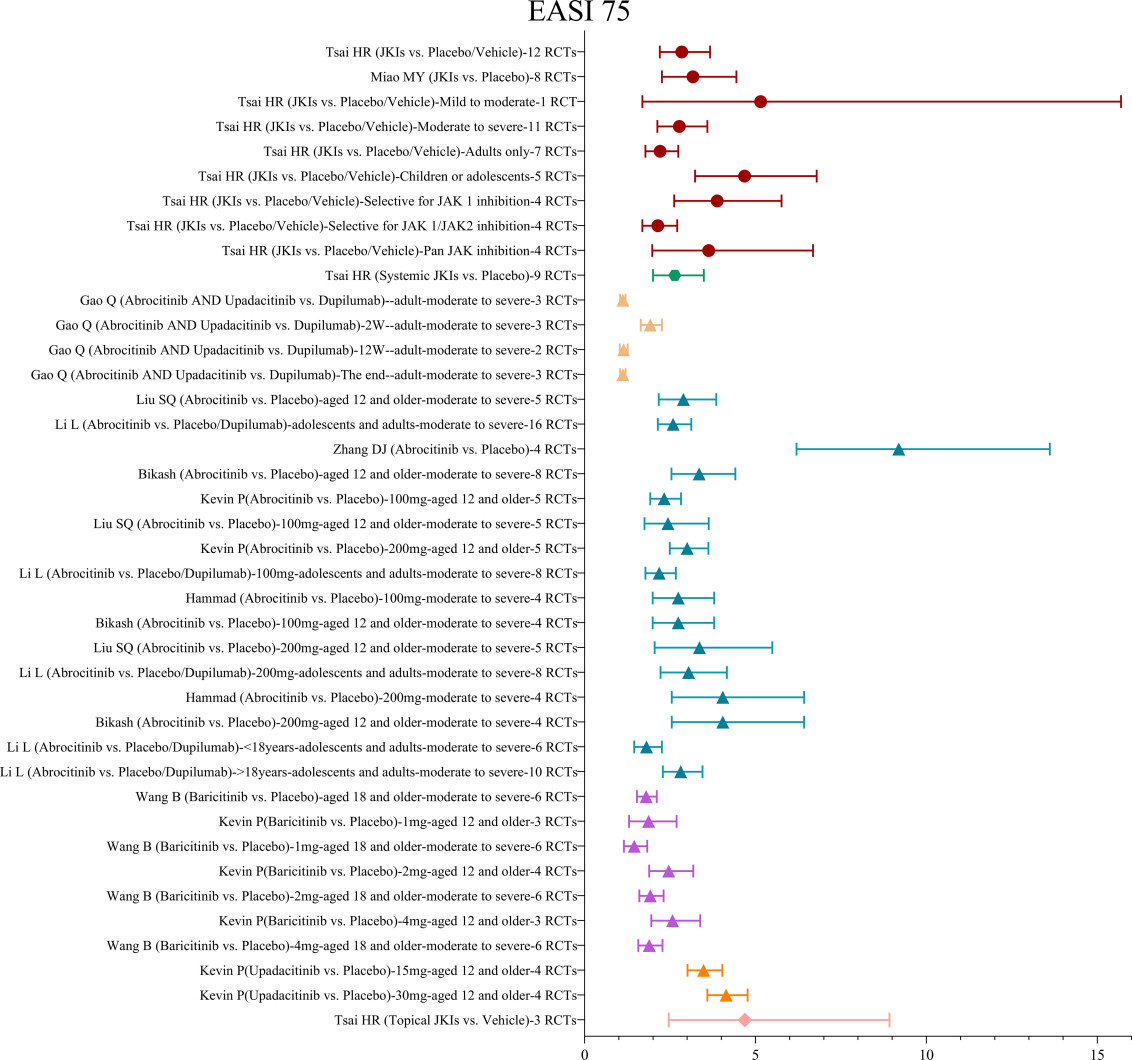
The summary evidence of the EASI75.

Merely one MA (29) reported improvement in the EASI75 with topical JKIs and pointed out that topical JKIs, as compared with placebos, were associated with a significantly higher rate of improvement in the EASI75 (RR=4.69, 95% CI [2.46, 8.92]). The result is shown in **Figure 5**.

##### 3.4.1.3 PP-NRS

**Figure 6:**
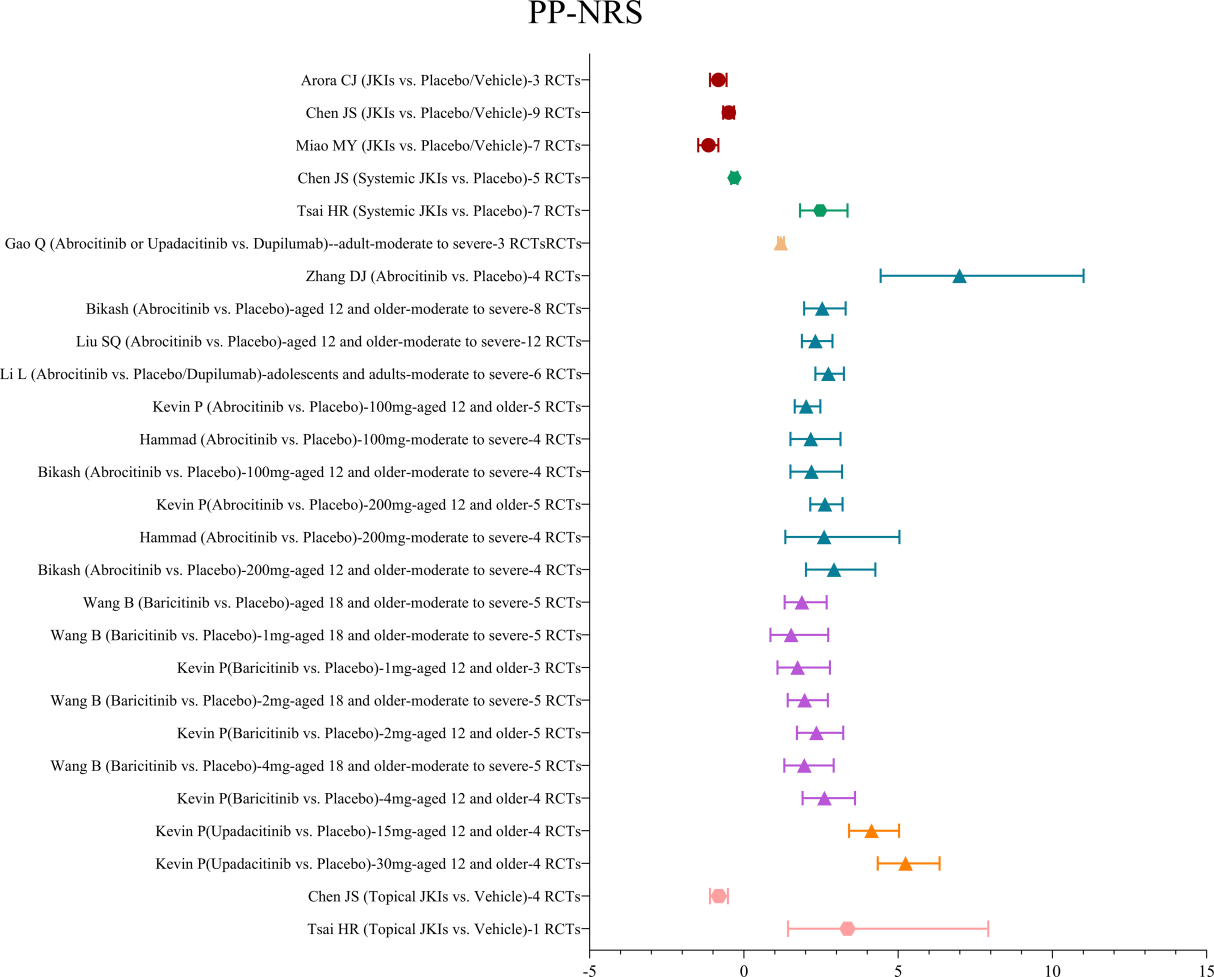
The summary evidence of the PP-NRS.

A total of 12 MAs examined the effect of JKIs on pruritus in patients with AD by the PP-NRS scale. Among these studies, three studies (28, 30, 31) used mean plus or minus standard deviation as the standardized mean difference and showed that JKIs were more active in reducing PP-NRS scores than placebos (SMD=-0.49, 95% CI [-0.67, 0.32]). Nine studies (19, 29, 33–39) evaluated the effect of ≥4 point improvement in the PP-NRS. With abrocitinib, baricitinib, and upadacitinib, the effect of ≥4 point improvement in the PP-NRS was significantly increased compared with placebos (RR=2.47, 95% CI [1.82, 3.36), while one study reported that 1 mg baricitinib did not show as superior versus placebo in moderate to severe AD (RR=1.53, 95% CI [0.86, 2.73]) (19). Similarly, according to one report (34), as compared with dupilumab, abrocitinib or upadacitinib was associated with slight amelioration of the PP-NRS (RR=1.2, 95% CI [1.11, 1.3). The details are shown in **Figure 6**.

#### 3.4.2 The safety of JKI inhibitors

##### 3.4.2.1 Adverse effects

**Figure 7:**
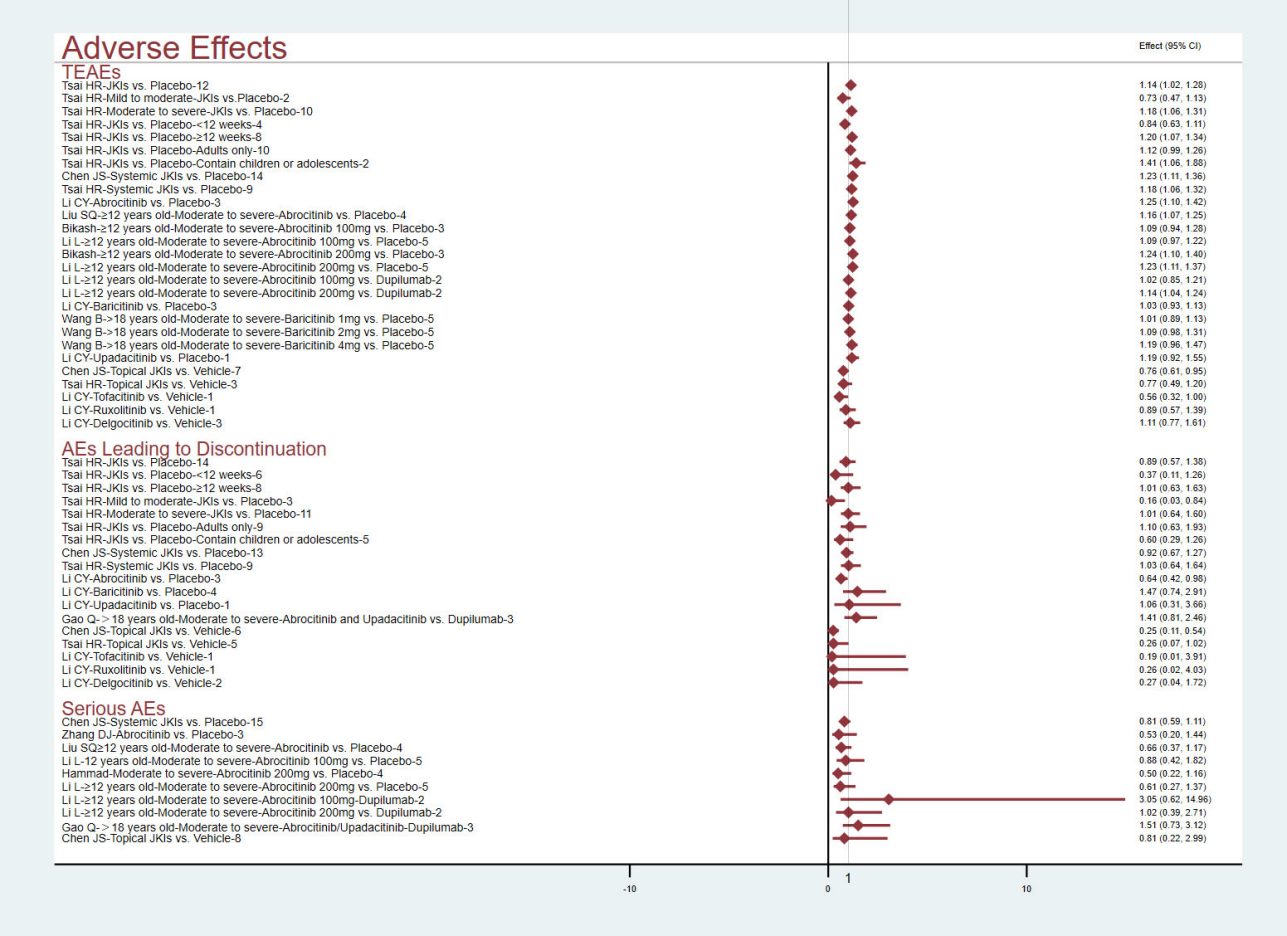
The summary evidence of adverse effects.

**Figure 7** conveys the details of the incidence of adverse effects. Pooled data from 10 RCTs showed that JKIs increased TEAEs versus placebos (RR=1.14, 95% CI [1.02, 1.28]) (29); after comparisons of several other studies that focused on systemic JKIs (RR=1.23, 95% CI [1.11, 1.36]) or topical JKIs (RR=0.76, 95% CI [0.61, 0.95]) alone, the results showed that the TEAEs were primarily derived from systemic JKIs (28). After subgroup analyses of the age of the participants, intervention duration, and AD severity, the results showed that the TEAEs from systemic JKIs came from those studies that included children or adolescents, had a duration more than 12 weeks, and moderate-to-severe AD (29). Whether compared with placebos (RR=1.23, 95% CI [1.11, 1.37]) or dupilumab (RR=1.14, 95% CI [1.04, 1.24]), 200 mg of abrocitinib led to more TEAEs in patients with moderate-to-severe AD. However, there was no statistically significant difference observed with 100 mg (P>0.05) (35, 38). Two MAs and one MA certified that baricitinib (RR=1.19, 95% CI [0.96, 1.47]) (19) and upadacitinib (RR=1.19, 95% CI [0.92, 1.55]) (17) would not increase TEAEs, respectively.

All MAs affirmed that, compared to placebos, JKI inhibitors did not increase adverse effects leading to discontinuation or serious adverse events (P>0.05).

##### 3.4.2.2 Skin diseases

Sanghyuk (22) addressed that abrocitinib had a higher risk of acne (RR=5.15, 95% CI [1.43, 18.57); further evidence suggested that this risk stems more directly from 200 mg of abrocitinib, regardless against placebo (RR=4.34, 95% CI [1.61, 11.71) or dupilumab (RR=4.59, 95% CI [2.6, 8.09]) (35). Current studies demonstrated that baricitinib did not increase the risk of acne (22). As for upadacitinib, one MA that pooled data from five RCTs emphasized its risk of causing acne at both 15mg (RR=3.93, 95% CI[2.54, 6.11]) and 30mg (RR=6.23, 95% CI [4.08, 9.49]) (22).

Results of one comprehensive study suggested that abrocitinib (RR=1.64, 95% CI [0.42, 6.39]), baricitinib (RR=1.77, 95% CI [0.47, 6.64]), and upadacitinib (RR=2.23, 95% CI [0.91, 5.47]) are not associated with a higher incident of herpes zoster (22).

**Figure 8:**
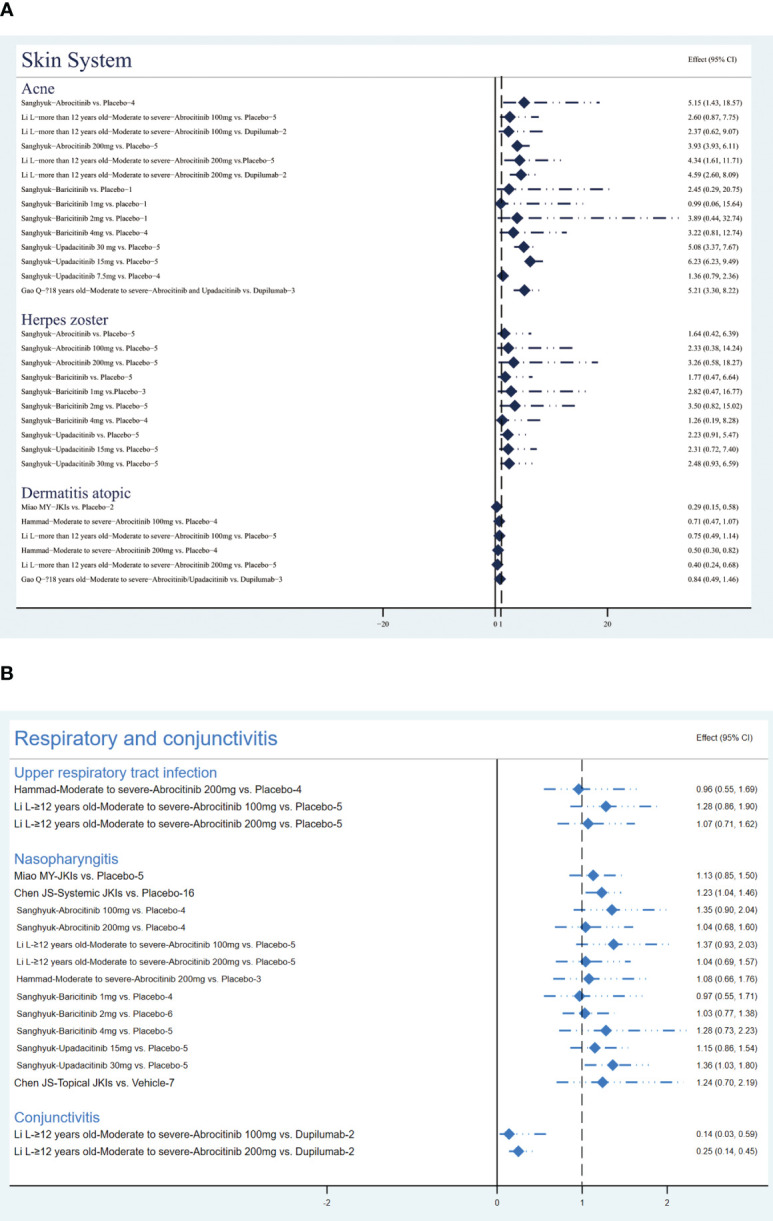
The summary evidence of **(A)** the skin system and **(B)** respiratory infections and conjunctivitis.

Three studies (30, 35, 39) revealed that abrocitinib at 200mg (RR=0.4, 95% CI [0.24, 0.68]) may be associated with reduced atopic dermatitis. The summary results are presented in **Figure 8A**.

##### 3.4.2.3 Respiratory infections and conjunctivitis

**Figure 8B** shows the results of respiratory infections and conjunctivitis events. The result from two MAs (35, 39) showed there was no statistically significant difference between the placebo and abrocitinib in the occurrence of upper respiratory tract infection events (P>0.05).

The two MAs disagreed on the occurrence of nasopharyngitis events; Miao MY suggested that JKIs were not associated with more nasopharyngitis events by pooling data from five RCTs (RR=1.13, 95% CI [0.85, 1.5]) (30), while Chen JS concluded, based on the results of 16 RCTs, that JKIs cause more nasopharyngitis events compared to placebo (RR=1.23, 95% CL [1.04, 1.46]) (28). In addition, three studies have demonstrated that abrocitinib and baricitinib did not increase nasopharyngitis (22, 35, 39) but 30 mg of upadacitinib did (RR=1.36, 95% CI [1.03, 1.8]) (22).

Li L (35) pooled two RCTs and suggested that compared to dupilumab, abrocitinib significantly decreased the incidence of conjunctivitis (RR=0.25, 95% [0.14, 0.45]).

##### 3.4.2.4 Nervous system

**Figure 9:**
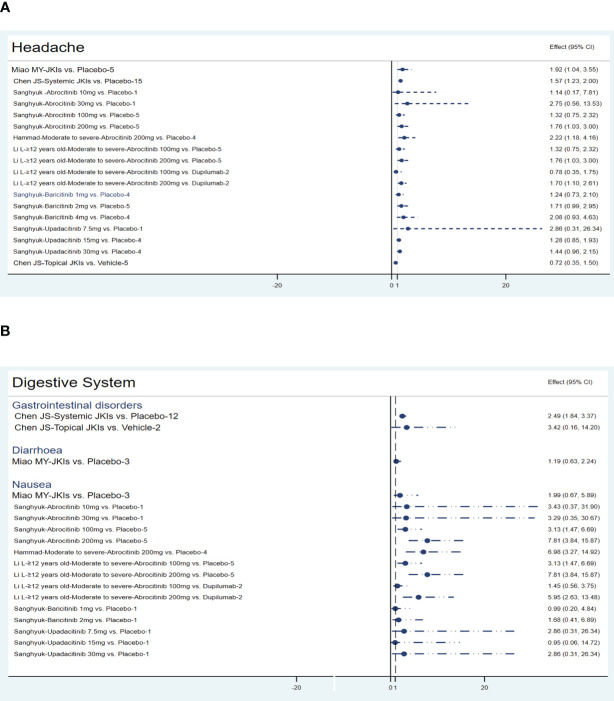
The summary evidence of **(A)** the nervous system and **(B)** the digestive system.

Five MAs (22, 28, 30, 35, 39) summarized the data on headaches. Miao (RR=1.92, 95% CI [1.04, 3.55], N=5) and Chen (RR=1.57, 95% CI [1.23, 2], N=15) highlighted that JKIs increase the risk of headache. Compared to placebo (RR=1.76, 95% CI [1.03, 3]) or dupilumab (RR=1.7, 95% CI [1.1, 2.61]), 200mg of abrocitinib causes more headaches. Furthermore, baricitinib, upadacitinib, and topical JKIs did not add more headache cases. The results are listed in **Figure 9A**.

##### 3.4.2.5 Digestive system

Compared to placebos, systemic JKIs increase the incidence of gastrointestinal disorders (RR=2.49, 95% [1.84, 3.37]) (28) without raising diarrhea (30). The three MAs concurrently yielded an increased nausea response in abrocitinib (100mg or 200mg) (22, 39), but this was not apparent in either baricitinib or upadacitinib (P>0.05) (22). The details are shown in **Figure 9B**.

##### 3.4.2.6 Cardiovascular events

By analyzing data from five RCTs, Sanghyuk (22) noted that major adverse cardiovascular events (MACEs) may occur with the application of baricitinib (RR=1.52, (95% CI [0.06, 38.86]) and upadacitinib (RR=2.97,95% CI [0.12, 38.86]), but the difference was not statistically significant compared with the placebo group. The use of abrocitinib, baricitinib, upadacitinib, and SHR0302 may be associated with an increase in thromboembolism, but there is no statistically significant difference (22, 32).

**Figure 10:**
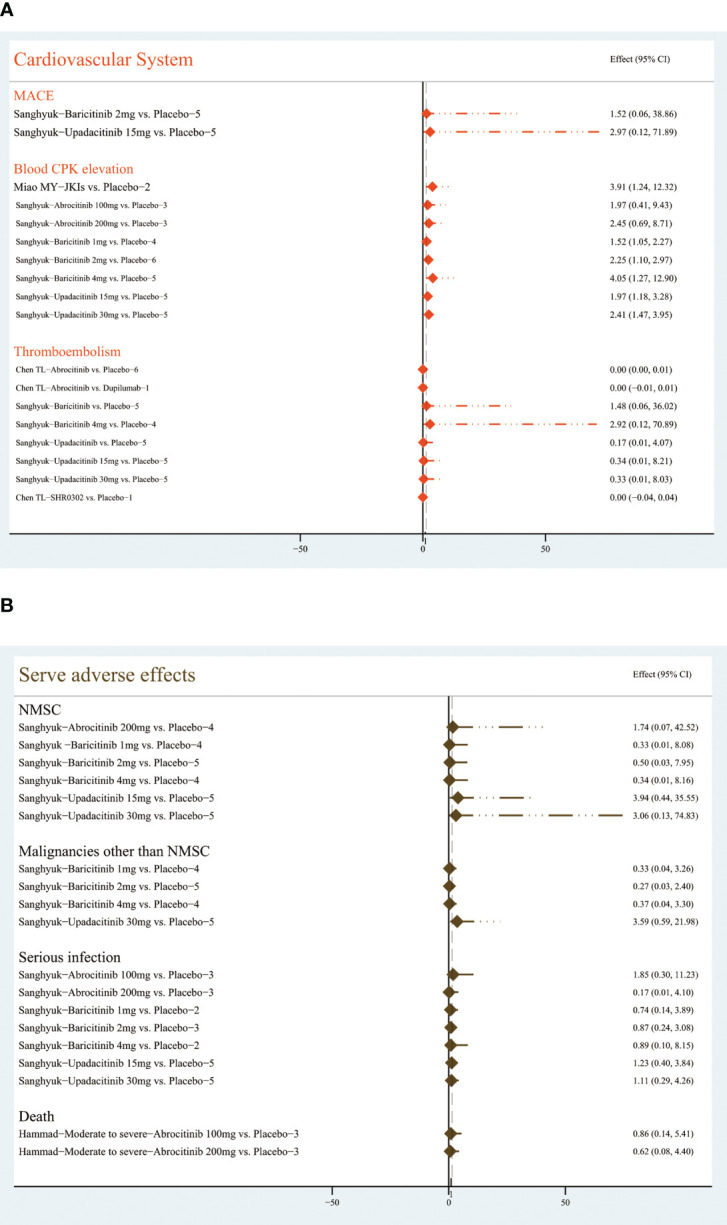
The summary evidence of **(A)** the cardiovascular system and **(B)** severe adverse effects.

Miao MY (30) summarized data from three RCTs, stating that JKIs cause increased blood creatine phosphokinase (RR=3.91, 95% CI [1,24, 12.32]). Sanghyuk (22) further noted that baricitinib at 2mg (RR=2.25, 95% CI [1.1, 2.97]) or 4mg (RR=4.05, 95% CI [1.27, 12.9]) and upadacitinib at 15mg (RR=1.97, 95%CI [1.18, 3.28]) or 30mg (RR=2.41, 95% CI [1.47, 3.95]) could be major contributors. **Figure 10A** shows the details.

##### 3.4.2.7 Serve AEs

Sanghyuk (22) focused on the safety of abrocitinib, baricitinib, and upadacitinib, and the results showed that the risk of serious infection, non-melanoma skin cancer (NMSC), and malignancies other than NMSC was not increased. Hammad (39) pooled three RCTs and revealed no difference between 100 or 200 mg abrocitinib and placebo on the risk of death (RR=0.86; 95% CI: [0.14, 5.41]) (RR=0.62, 95% CI [0.08, 4.4]), respectively. The **Figure 10B** lists the results.

### 3.5 Certainty of main evidence based on GRADE

Based on the original data of MAs that we can obtain, we used GRADE to evaluate the credibility of the evidence of IGA response, the EASI75, and the PP-NRS. The results suggested that the levels of evidence for IGA response was “high” regardless of JKIs, systemic JKI, topical JKI, abrocitinib, or baricitinib. Similarly, the levels of evidence for most results on the EASI75 were “high quality” except for topical JKIs (moderate-quality evidence). As for the effect of abrocitinib on the PP-NRS, two MAs (36, 38) indicated that the certainty of evidence was “high”; nevertheless, another study (39) proved that the level of evidence quality was “very low”, mainly considering the risk of bias, inconsistency, and imprecision. The findings are summarized in **Supplementary Table 3**, which describes the complete assessment with footnotes explaining each judgment.

## 4 Discussion

### 4.1 Summary of main results

This umbrella review incorporates 16 MAs which evaluated the efficacy and safety of JKIs for AD. Three of these focused only on safety indicators, one evaluated efficacy indicators, and the remaining 12 studies (four for JKIs, two for systemic JKIs, five for abrocitinib, and one for baricitinib) evaluated both safety and efficacy.

The available evidence suggests that, compared to placebos or dupilumab, the administration of JKIs can ameliorate IGA response more effectively, (high-quality evidence), improve the EASI75 (high-quality evidence), and relieve pruritus without severe adverse effects (adverse cardiovascular events, tumors, serious infections, or death).

Topical JKIs have demonstrated superior therapeutic efficacy, although the number of studies was limited. This study revealed no disparity in the effectiveness of systemic JKIs for AD across different severities. However, compared to those involving adults only, JKIs exhibited greater efficacy in studies involving both adolescents and adults. Regarding specific drugs, the findings indicated that 200mg of abrocitinib outperformed 100mg, with significant results observed at 4 weeks. Additionally, 2mg and 4mg of baricitinib were more effective than 1mg in improving IGA response and the EASI75. Abrocitinib demonstrated effectiveness, albeit in a limited number of studies; however, it may lead to digestive disturbances, nausea, headaches, and an increased likelihood of acne and herpes zoster in AD patients. Notably, the likelihood of experiencing these conditions was higher with a dosage of 200mg compared to 100mg abrocitinib. Baricitinib, particularly at a dosage of 2mg, can cause elevated blood CPK. Similarly, the usage of upadacitinib also elevated blood CPK and was associated with an increased incidence of nasopharyngitis and skin acne.

Based on the results of AMSTAR-II, 12.5% of the studies were judged to be of “high” methodological quality and 87.5% were of “moderate” quality (due to non-registration, non-reporting of funding sources for RCTs, and consolidation of data without considering the methodological quality of RCTs). Generally, we can be confident of the methodological quality of these systematic reviews.

### 4.2 Overall completeness and applicability of evidence

It is widely recognized that atopic dermatitis is a complex skin disease involving multiple dimensions such as genetics, immunity, allergies, and inflammation (40–42). To date, in the field of dermatology, atopic dermatitis has become an increasingly prevalent problem that must be tackled (2, 3, 43). Many key cytokines involved in the pathogenesis of atopic dermatitis, such as IL-4, IL-13, IL-31, and TSLP, initiate intracellular signaling through the Janus tyrosine kinase and signal transducer and activator of transcription pathway (44), leading to the development of skin lesions and itching in patients. Janus, meaning “two-faced god” in ancient Roman mythology, was a gatekeeper (45, 46). Interestingly, JKIs, like gatekeepers, regulate the entry and exit of inflammatory pathways because of the important role of the JAK/STAT pathway in the pathogenesis of atopic dermatitis. Unlike biologics with a relatively single target, such as anti-interleukin agents, JKIs overcome the “limitations” of precise targeting, blocking a variety of signal transmission involved in immune response and inflammatory factors (47). Specifically, JKIs have an active effect on atopic dermatitis by blocking the JAK-STAT signaling pathway mediated by IL-4, IL-13, IL-31, and other related cytokines, which affects the expression of downstream genes (48–50). To date, four members of the JAK family have been identified: JAK1, JAK2, JAK3, and TYK2. Among them, JAK1 is associated with inflammation, immune diseases, and tumors, JAK2 is related to hematological diseases, and JAK3 is relevant to autoimmune diseases (51). AD patients treated with JKIs can rapidly relieve itching and reduce inflammation-related symptoms such as exudation, redness, and swelling (52). Hence, IGA response, the EASI75, and the PP-NRS were significantly improved after JKI treatment.

JAK inhibitors, which alleviate the inflammatory symptoms of atopic dermatitis by blocking the JAK/STAT pathway, can also hinder the transmission of vital cytokines in the body. This is especially true when JAK inhibitors are not accurately targeted, disrupting overall cytokine expression in the body and interfering with other signaling pathways, leading to a series of unfavorable effects. Blood creatinine phosphokinase elevation is an adverse effect of 2 mg baricitinib and 15 mg upadacitinib, as well as a characterization of cardiovascular events. JKIs activate mTORC1 by inhibiting the STAT pathway (53); this may be a potential mechanism for the occurrence of CPK elevation. Follicular keratosis is the main causative factor of acne (54), and JKls can cross-talk epidermal growth factor signaling through the JAK/STAT pathway to cause aberrant follicular keratinization (55), which may contribute to the prevalence of acne in abrocitinib and upadacitinib. As for the nasopharyngitis that accompanied upadacitinib, the exact mechanism of this effect is not clear; however, we consider that over-activation of the inflammatory response by JKIs may be the main reason. Our findings indicated that abrocitinib was linked to a rise in occurrences of headache and nausea, potentially due to the interference of the JAK/STAT pathway with the normal operations of the central nervous system (56, 57). Thus, the clinical use and future development of JKIs might need to balance its effects on immunological networks (58), and highly selective inhibitors may be one of the development directions for JKIs.

### 4.3 Agreements and disagreements with other studies

Our study is the first umbrella review of systematic review of JKIs for atopic dermatitis. Since the appearance of the first systematic review of JKIs in 2020, subsequent systematic reviews or reviews have emphasized the therapeutic potential of JAK inhibitors for AD, either expanding the sample size, evaluating topical or systemic JKIs separately (33, 59–61), or concentrating on the safety of JKIs (62); the evidence is diverse and the quality of the methodologies is uncertain. Our study presented a straightforward overview of all currently published meta-analyses on JKIs and assessed the quality of the evidence.

We observed that there were numerous network meta-analyses conducted to compare the variations in effectiveness and safety among various medications, diverse methods of administration, and distinct age groups within the population. In line with our findings, Alexandro stressed that upadacitinib and high-dose abrocitinib were effective but also among the most “alarming” (63). However, in contrast with a comprehensive meta-analysis conducted by Axel which revealed that upadacitinib was more effective in adults than in adolescents (64), our research indicated that JKIs may have a greater impact on children or adolescents; thus, it is recommended to tailor the use of JKIs to specific age groups. Undoubtedly, there is great concern regarding the safety of JKIs due to their correlation with serious adverse events; in July 2022, the FDA added a “black box warning” for upadacitinib, tofacitinib, and baricitinib, and patients were also cautioned that JKIs may increase the risk of heart disease, tumors, venous thrombosis, and death (65, 66). Additionally, the latest data integration results indicated that JKIs are more prone to causing skin inflammation, upper respiratory tract infections, and gastrointestinal adverse reactions when compared to placebos.

A real-world study, albeit with a small sample size (n=41), demonstrated that abrocitinib was effective in reducing both EASI75 and PP-NRS scores on patients with difficult-to-treat atopic dermatitis, although along with nausea, acne and respiratory tract infection. (67). One real-world study that included 48 difficult-to-treat atopic dermatitis patients showed that upadacitinib was an effective treatment and 14 case adverse effects were reported, including acne-like eruptions, nausea, and respiratory tract infections (68). Such evidence from the real world once again corroborated our findings.

### 4.4 Limitations and suggestions for future studies

Considering the methodological aspects, we noticed that over 85% of the MAs were rated with moderate confidence in the findings of the AMSTAR-2 evaluation. Being unable to establish a study protocol, not assessing and discussing the possible impact of the risk of primary study bias, and lacking the reporting of the source of funding for the original study were the major issues. In an effort to generate more credible and higher-quality evidence, protocol registration is the first step in meta-analysis, and the impact of funding grants and related bias should be fully considered when conducting the analysis of MA data.

By and large, the quality of evidence was “high”, demonstrating that the results of IGA response and the EASI75 were credible, whereas the level of evidence for the PP-NRS was divisive; low evidence quality was mainly attributed to the risk of bias, inconsistency, and imprecision. Thus, it is important to focus on selecting high-quality original studies for review, and more attention should be given to the effect of abrocitinib in PP-NRS.

In terms of content, we note that the evidence for abrocitinib is the most comprehensive; more clinical studies are needed to explore the effects of topical JKIs and the effects of baricitinib and upadacitinib in patients with atopic dermatitis. In addition, considering that the therapeutic benefits of high-dose drugs are often accompanied by more adverse events, precise treatment using JKIs in different populations and of treatment durations is the main direction of future research. Moreover, a positive correlation between severe adverse events and JKI inhibitors was not found statistically, which may require more data from real-world sources and randomized controlled trials.

## 5 Conclusion

Compared to placebos or dupilumab, the administration of JKIs can ameliorate IGA response more effectively, improve the EASI75, and relieve pruritus without severe adverse effect, while accompanied by more acne, nasopharyngitis, headache, and digestive disturbances. In the application of abrocitinib, 200 mg is the most efficient, but it should be used with caution in patients with gastrointestinal dysfunction, herpes zoster, and those who are acne-prone. Baricitinib and upadacitinib should be avoided in populations at high risk for cardiovascular events.

## Data availability statement

The original contributions presented in the study are included in the article/**Supplementary Material**. Further inquiries can be directed to the corresponding authors.

## Author contributions

QH: Data curation, Formal analysis, Investigation, Methodology, Visualization, Writing – original draft, Writing – review & editing. XX: Formal analysis, Investigation, Visualization, Writing – original draft. QC: Formal analysis, Investigation, Methodology, Visualization, Writing – review & editing. WL: Data curation, Investigation, Writing – original draft. ZS: Methodology, Visualization, Writing – original draft. XW: Data curation, Formal analysis, Writing – original draft. XM: Conceptualization, Project administration, Writing – review & editing. JZ: Conceptualization, Project administration, Writing – original draft. JG: Funding acquisition, Project administration, Supervision, Writing – review & editing.
