# Supplementary material for: Janus kinase inhibitors in atopic dermatitis: an umbrella review of meta-analyses
Source: Front Immunol. 2024 Feb 23;15:1342810. doi: 10.3389/fimmu.2024.1342810 (PMC10921355; doi:10.3389/fimmu.2024.1342810)
Supplement: Supplementary file 1 [file Table_1.docx]

**Supply table 1. Literature search strategy**

| **The database** | **Retrieval type** | **Result** |
| --- | --- | --- |
| **Pubmed** | **(((dermatitis, atopic[MeSH Terms]) OR (((((((((((((((Atopic Dermatitides[Title/Abstract]) OR (****Atopic Dermatitis[Title/Abstract])) OR (Dermatitides, Atopic[Title/Abstract])) OR (Neurodermatitis, Atopic[Title/Abstract])) OR (Atopic Neurodermatitides[Title/Abstract])) OR (Atopic Neurodermatitis[Title/Abstract])) OR (Neurodermatitides, Atopic[Title/Abstract])) OR (Neurodermatitis, Disseminated[Title/Abstract])) OR (Disseminated Neurodermatitides[Title/Abstract])) OR (Disseminated Neurodermatitis[Title/Abstract])) OR (Neurodermatitides, Disseminated[Title/Abstract])) OR (Eczema, Atopic[Title/Abstract])) OR (Atopic Eczema[Title/Abstract])) OR (Eczema, Infantile[Title/Abstract])) OR (Infantile Eczema[Title/Abstract]))) AND (((((((((((((((((Janus Kinase Inhibitors[MeSH Terms]) OR (((((((((Inhibitors, Janus Kinase[Title/Abstract]) OR (Kinase Inhibitors, Janus[Title/Abstract])) OR (JAK Inhibitors[Title/Abstract])) OR (Inhibitors, JAK[Title/Abstract])) OR (Janus Kinase Inhibitor[Title/Abstract])) OR (Inhibitor, Janus Kinase[Title/Abstract])) OR (Kinase Inhibitor, Janus[Title/Abstract])) OR (JAK Inhibitor[Title/Abstract])) OR (Inhibitor, JAK[Title/Abstract]))) OR (((((abrocitinib[Title/Abstract]) OR (PF-04965842[Title/Abstract])) OR ("N-(cis-3-(Methyl(7H-pyrrolo(2,3-d)pyrimidin-4-yl)amino)cyclobutyl)-1-propanesulfonamide"[Text Word])) OR ("N-(cis-3-(Methyl(7H-pyrrolo(2,3-d)pyrimidin-4-yl)amino)cyclobutyl)-propane-1-sulfonamide"[Text Word])) OR ("1-Propanesulfonamide, N-(cis-3-(methyl-7H-pyrrolo(2,3-d)pyrimidin-4-ylamino)cyclobutyl)"[Text Word]))) OR ((((upadacitinib[Title/Abstract]) OR (ABT-494[Title/Abstract])) OR (Rinvoq[Title/Abstract])) OR ("(3S,4R)-3-ethyl-4-(3H-imidazo(1,2-a)pyrrolo(2,3-e)pyrazin-8-yl)-N-(2,2,2-trifluoroethyl)-1-pyrrolidinecarboxamide"[Text Word]))) OR ((((((((((((((((ruxolitinib[Title/Abstract]) OR (ruxolitinib phosphate[Title/Abstract])) OR (ruxolitinib monophosphate[Title/Abstract])) OR (Jakavi[Title/Abstract])) OR (Jakafi[Title/Abstract])) OR (INCB-18424 phosphate[Title/Abstract])) OR (INCB018424 phosphate[Title/Abstract])) OR (INCB-018424 salt[Title/Abstract])) OR (INCB-018424 phosphate[Title/Abstract])) OR (INCB-018424[Title/Abstract])) OR (INC-424[Title/Abstract])) OR (INCB-18424[Title/Abstract])) OR (INC424[Title/Abstract])) OR (INCB018424[Title/Abstract])) OR (INCA24[Title/Abstract])) OR ("3R-3-cyclopentyl-3-(4-(7H-pyrrolo(2,3-d)pyrimidin-4-yl)pyrazol-1-yl)propanenitrile"[Text Word]))) OR (((((((((((baricitinib[Title/Abstract]) OR (Olumiant[Title/Abstract])) OR (baricitinib phosphate[Title/Abstract])) OR (baricitinib phosphate salt[Title/Abstract])) OR (INCB-28050[Title/Abstract])) OR (INCB028050[Title/Abstract])) OR (INCB-028050[Title/Abstract])) OR (LY3009104[Title/Abstract])) OR (LY-3009104[Title/Abstract])) OR ("3-azetidineacetonitrile, 1-(ethylsulfonyl)-3-(4-(7H-pyrrolo(2,3-d)pyrimidin-4-yl)-1H-pyrazol-1-yl)"[Text Word])) OR ("3-azetidineacetonitrile, 1-(ethylsulfonyl)-3-(4-(7H-pyrrolo(2,3-d)pyrimidin-4-yl)-1H-pyrazol-1-yl)-, phosphate (1:1)"[Text Word]))) OR ((((delgocitinib[Title/Abstract]) OR (JTE-052[Title/Abstract])) OR ("3-((3S,4R)-3-methyl-6-(7H-pyrrolo(2,3-d)pyrimidin-4-yl)-1,6-diazaspiro(3.4)octan-1-yl)-3-oxopropanenitrile"[Text Word])) OR ("1,6-Diazaspiro(3.4)octane-1-propanenitrile, 3-methyl-beta-oxo-6-(7H-pyrrolo(2,3-d)pyrimidin-4-yl)-, (3S,4R)-"[Text Word]))) OR (((((((((Tofacitinib[Title/Abstract]) OR (tasocitinib[Title/Abstract])) OR (tofacitinib citrate[Title/Abstract])) OR (Xeljanz[Title/Abstract])) OR (CP 690,550[Title/Abstract])) OR (CP690550[Title/Abstract])) OR (CP-690550[Title/Abstract])) OR (CP 690550[Title/Abstract])) OR (CP-690,550[Title/Abstract]))) OR (((((((((((((fedratinib[Title/Abstract]) OR (Inrebic[Title/Abstract])) OR (fedratinib hydrochloride[Title/Abstract])) OR (fedratinib hydrochloride monohydrate[Title/Abstract])) OR (fedratinib dihydrochloride monohydrate[Title/Abstract])) OR (TG101348[Title/Abstract])) OR (TG-101348[Title/Abstract])) OR (SAR302503[Title/Abstract])) OR (SAR-302503A[Title/Abstract])) OR (SAR302503A[Title/Abstract])) OR (SAR-302503[Title/Abstract])) OR ("N-tert-butyl-3-(5-methyl-2-(4-(2-(pyrrolidin-1-yl)ethoxy)phenylamino) pyrimidin-4-ylamino)benzenesulfonamide"[Text Word])) OR ("N-tert-butyl-3-((5-methyl-2-((4-(2-pyrrolidin-1-ylethoxy)phenyl)amino)pyrimidin-4-yl)amino) benzenesulfonamide dihydrochloride monohydrate"[Text Word]))) OR ((((gilteritinib[Title/Abstract]) OR (Xospata[Title/Abstract])) OR (ASP-2215[Title/Abstract])) OR (ASP2215[Title/Abstract]))) OR ((((peficitinib[Title/Abstract]) OR (ASP015K[Title/Abstract])) OR ("4-((trans-5-hydroxyadamantan-2-yl)amino)-1H-pyrrolo(2,3-b)pyridine-5-carboxamide"[Text Word])) OR ("4-((5-hydroxy-2-adamantyl)amino)-1H-pyrrolo(2,3-b)pyridine-5-carboxamide"[Text Word]))) OR ((GLPG0634[Title/Abstract]) OR (filgotinib[Title/Abstract]))) OR (((((pacritinib[Title/Abstract]) OR (SB 1518[Title/Abstract])) OR (SB1518[Title/Abstract])) OR (SB-1518[Title/Abstract])) OR ("11-(2-pyrrolidin-1-ylethoxy)-14,19-dioxa-5,7,26-triazatetracyclo(19.3.1.1(2,6).1(8,12))heptacosa-1(25),2(26),3,5,8,10,12(27),16,21,23-decaene"[Text Word]))) OR ((oclacitinib[Title/Abstract]) OR (apoquel[Title/Abstract]))) OR (((((Momelotinib[Title/Abstract]) OR (CYT 387[Title/Abstract])) OR (CYT387[Title/Abstract])) OR (CYT-387[Title/Abstract])) OR ("N-(cyanomethyl)-4-(2-((4-(4-morpholinyl)phenyl)amino)-4-pyrimidinyl)benzamide"[Text Word]))) OR ((itacitinib[Title/Abstract]) OR ("(1-(1-(3-Fluoro-2-(trifluoromethyl)isonicotinoyl)-4-piperidinyl)-3-(4-(1H-pyrrolo(2,3-d)pyrimidin-4-yl)-1H-pyrazol-1-yl)-3-azetidinyl)acetonitrile"[Text Word]))) OR (Jaktinib[Title/Abstract]))) AND (((((Systematic Review[Publication Type]) OR (Systematic Reviews as Topic[MeSH Terms])) OR (Systematic Review[Title/Abstract])) OR ((((Meta-Analysis[Publication Type]) OR (Meta-Analysis as Topic[MeSH Terms])) OR (meta analyses[Title/Abstract])) OR (meta analysis[Title/Abstract]))) OR ((((Review[Publication Type]) OR (Review Literature as Topic[MeSH Terms])) OR (review[Title/Abstract])) OR (overview[Title/Abstract])))** | **294** |
| **Embase** | **#1** 'atopic dermatitis'/exp OR 'dermatitis, atopic':ab OR 'atopic dermatitides':ab OR 'dermatitides, atopic':ab OR 'neurodermatitis, atopic':ab OR 'atopic neurodermatitides':ab OR 'atopic neurodermatitis':ab OR 'neurodermatitides, atopic':ab OR 'neurodermatitis, disseminated':ab OR 'disseminated neurodermatitides':ab OR 'disseminated neurodermatitis':ab OR 'neurodermatitides, disseminated':ab OR 'eczema, atopic':ab OR 'atopic eczema':ab OR 'eczema, infantile':ab OR 'infantile eczema':ab  **#2** 'janus kinase inhibitor'/exp OR 'janus kinase inhibitor' OR (('janus'/exp OR janus) AND ('kinase'/exp OR kinase) AND ('inhibitor'/exp OR inhibitor)) OR 'janus kinase 1 inhibitor'/exp OR 'janus kinase 2 inhibitor'/exp OR 'janus kinase 3 inhibitor'/exp OR (4:ab,ti AND '3 bromo 4 hydroxyanilino':ab,ti AND '6,7 dimethoxyquinazoline':ab,ti) OR atinvicitinib:ab,ti OR baricitinib:ab,ti OR brepocitinib:ab,ti OR delgocitinib:ab,ti OR deucravacitinib:ab,ti OR deuruxolitinib:ab,ti OR fedratinib:ab,ti OR fosifidancitinib:ab,ti OR gusacitinib:ab,ti OR ifidancitinib:ab,ti OR ilginatinib:ab,ti OR ilunocitinib:ab,ti OR itacitinib:ab,ti OR izencitinib:ab,ti OR lorpucitinib:ab,ti OR mivavotinib:ab,ti OR momelotinib:ab,ti OR ('n benzyl 2 cyano 3':ab,ti AND '3,4 dihydroxyphenyl':ab,ti AND acrylamide:ab,ti) OR nezulcitinib:ab,ti OR oclacitinib:ab,ti OR ropsacitinib:ab,ti OR ruxolitinib:ab,ti OR tofacitinib:ab,ti OR upadacitinib:ab,ti OR abrocitinib:ab,ti OR filgotinib:ab,ti OR golidocitinib:ab,ti OR ivarmacitinib:ab,ti OR povorcitinib:ab,ti  **#3** 'systematic review':ti OR 'reviews systematic':ti OR 'meta analysis':ti OR 'meta analyses':ti OR review:ti OR overview:ti  **#1 AND #2 AND #3** | **157** |
| **Web of Science** | **#1** (((((((((((((((TS=("dermatitis, atopic")) OR TI=(Atopic dermatitidis)) OR TI=(Atopic Dermatitis)) OR TI=("dermatitidis, Atopic")) OR TI=("dermatitidis, Atopic")) OR TI=(Atopic neurodermatitis)) OR TI=(Atopic Neurodermatitis)) OR TI=("neurodermatitis, Atopic")) OR TI=("Neurodermatitis, Disseminated")) OR TI=(Disseminated neurodermatitis)) OR TI=(Disseminated Neurodermatitis)) OR TI=("neurodermatitis, Disseminated")) OR TI=("Eczema, Atopic")) OR TI=(Atopic Eczema)) OR TI=("Eczema, Infantile")) OR TI=(Infantile Eczema)  **#2** (((((((((((((((((((((((((((((((TS=(Janus Kinase Inhibitors)) OR AB=(Janus kinase 1 inhibitor)) OR AB=(Janus kinase 2 inhibitor)) OR AB=(Janus kinase 3 inhibitor)) OR AB=(atinvicitinib)) OR AB=(baricitinib)) OR AB=(brepocitinib)) OR AB=(delgocitinib)) OR AB=(deucravacitinib)) OR AB=(deuruxolitinib)) OR AB=(fedratinib)) OR AB=(fosifidancitinib)) OR AB=(gusacitinib)) OR AB=(ifidancitinib)) OR AB=(ilginatinib)) OR AB=(ilunocitinib)) OR AB=(itacitinib)) OR AB=(izencitinib)) OR AB=(lorpucitinib)) OR AB=(mivavotinib)) OR AB=(momelotinib)) OR AB=(nezulcitinib)) OR AB=(oclacitinib)) OR AB=(ropsacitinib)) OR AB=(ruxolitinib)) OR AB=(tofacitinib)) OR AB=(upadacitinib)) OR AB=(abrocitinib)) OR AB=(filgotinib)) OR AB=(golidocitinib)) OR AB=(ivarmacitinib)) OR AB=(povorcitinib)  **#3** ((((((TI=(systematic review)) OR TI=(reviews systematic)) OR TI=(meta analysis)) OR TI=(meta analyses)) OR TI=(review)) OR TI=(overview)) OR TI=(cochrane review)  **#1 AND #2 AND #3** | **70** |
| **Cochrane Library** | **#1** (dermatitis, atopic OR Atopic dermatitidis OR Atopic Dermatitis OR dermatitidis, Atopic OR dermatitidis, Atopic OR Atopic neurodermatitis OR Atopic Neurodermatitis OR neurodermatitis, Atopic OR Neurodermatitis, Disseminated OR Disseminated neurodermatitis OR Disseminated Neurodermatitis OR neurodermatitis, Disseminated OR Eczema, Atopic OR Atopic Eczema OR Eczema, Infantile OR Infantile Eczema):ti,ab,kw  **#2** (Janus Kinase Inhibitors OR Janus kinase 1 inhibitor OR Janus kinase 2 inhibitor OR Janus kinase 3 inhibitor OR atinvicitinib OR baricitinib OR brepocitinib OR delgocitinib OR deucravacitinib OR deuruxolitinib OR fedratinib OR fosifidancitinib OR gusacitinib OR ifidancitinib OR ilginatinib OR ilunocitinib OR itacitinib OR izencitinib OR lorpucitinib OR mivavotinib OR momelotinib OR nezulcitinib OR oclacitinib OR ropsacitinib OR ruxolitinib OR tofacitinib OR upadacitinib OR abrocitinib OR filgotinib OR golidocitinib OR ivarmacitinib OR povorcitinib):ti,ab,kw  **#3** (systematic review OR reviews systematic OR meta analysis OR meta analyses OR review OR overview OR cochrane review):ti,ab,kw  **#1 AND #2 AND #3** | **47** |
| **CNKI** | ((TKA=特应性皮炎) OR (TKA=异位性皮炎) OR (TKA=异位性湿疹) OR (TKA=特应性湿疹) OR (TKA=特异性皮炎) OR (TKA=遗传过敏性皮炎) OR (TKA=遗传过敏性湿疹) OR (TKA=四弯风) OR (TKA=儿湿疹)) AND ((FT=Janus激酶抑制剂) OR (FT=JAK抑制剂) OR (FT=Atinvicitinib) OR (FT=Baricitinib) OR (FT=Brepocitinib) OR (FT=Brepocitinib) OR (FT=delgocitinib) OR (FT=deucravacitinib) OR (FT=fosifidancitinib) OR (FT=fedratinib) OR (FT=gusacitinib) OR (FT=ifidancitinib) OR (FT=ilginatinib) OR (FT=ilunocitinib) OR (FT=itacitinib) OR (FT=izencitinib) OR (FT=lorpucitinib) OR (FT=mivavotinib) OR (FT=momelotinib) OR (FT=nezulcitinib) OR (FT=oclacitinib) OR (FT=ropsacitinib) OR (FT=ruxolitinib) OR (FT=ruxolitinib) OR (FT=upadacitinib) OR (FT=abrocitinib) OR (FT=filgotinib) OR (FT=golidocitinib) OR (FT=ivarmacitinib) OR (FT=povorcitinib)) AND ((TKA=系统评价) OR (TKA=系统综述) OR (TKA=meta分析) OR (TKA=荟萃分析) OR (TKA=汇总分析)) | **5** |
| **WanFang** | (题名或关键词:(特应性皮炎) or 题名或关键词:(异位性皮炎) or 题名或关键词:(异位性湿疹) or 题名或关键词:(异位性湿疹) or 题名或关键词:(特异性皮炎) or 题名或关键词:(遗传过敏性皮炎) or 题名或关键词:(遗传过敏性湿疹) or 题名或关键词:(儿湿疹) or 题名或关键词:(四弯风)) AND (全部:(Janus激酶抑制剂) or 全部:(JAK抑制剂) or 全部:(Atinvicitinib) or 全部:(Baricitinib) or 全部:(Brepocitinib) or 全部:(Brepocitinib) or 全部:(delgocitinib) or 全部:(deucravacitinib) or 全部:(fosifidancitinib) or 全部:(fedratinib) or 全部:(gusacitinib) or 全部:(ifidancitinib) or 全部:(ilginatinib) or 全部:(ilunocitinib) or 全部:(itacitinib) or 全部:(izencitinib) or 全部:(lorpucitinib) or 全部:(mivavotinib) or 全部:(momelotinib) or 全部:(nezulcitinib) or 全部:(oclacitinib) or 全部:(ropsacitinib) or 全部:(ruxolitinib) or 全部:(ruxolitinib) or 全部:(upadacitinib) or 全部:(abrocitinib) or 全部:(filgotinib) or 全部:(golidocitinib) or 全部:(ivarmacitinib) or 全部:(povorcitinib)) | **10** |

**Supply table 2. Specific reading criteria for GRADE factors**

| **Category** | **Item** | **Points** | **Criteria** | **Score** |
| --- | --- | --- | --- | --- |
| Downgrade quality of evidence | Risk of bias | -2 | Judged by the Cochrane risk of bias tool for randomized trials | |
|  |  |  | All or most (2/3) were judged to be “low risk” | no |
|  |  |  | The majority (2/3) judged it as “moderate risk” | -1 |
|  |  |  | Most (2/3) were judged to be “high risk” | -2 |
|  | Inconsistency | -2 | Judged on the basis of heterogeneity | |
|  |  |  | I^2^＜50% | No |
|  |  |  | 50%≤I^2^＜75% | -1 |
|  |  |  | I^2^≥75% | -2 |
|  | Indirectness | -2 | The evidence comes from indirect comparisons rather than direct comparisons  Differences between studies in population ethnicity, interventions, and outcome indicators | None: no  With one of them: -1  Both: -2 |
|  | Imprecision | -2 | 95% confidence interval CI across the equivalence line  Small total sample size (<300 cases), wide confidence interval | None: no  With one of them: -1  Both: -2 |
|  | Publication bias | -1 | Significant publication bias or research funded entirely by corporate grants | None: no  Yes: -1 |
| Upgrade quality of evidence | Large effect | +2 | 0.5 ≤ RR ≤ 2 | No |
|  |  |  | RR >2 or <0.5 (based on consistent evidence from at least 2 studies, with no plausible confounders) | +1 |
|  |  |  | RR >5 or <0.2 (based on direct evidence with no major threats to validity) | +2 |
|  | Plausible confounding would change the effect | +1 | Inclusion in the study took into account the effect of possible confounding factors on the results, and the actual effect was more pronounced than statistical | No  Yes: +1 |
|  | Dose-Response Gradient | +1 | Dose-Response Gradient | No  Yes: +1 |

**Supply table 3. Certainty of evidence based on GRADE**

| **JKIs compared to Placebo for Atopic Dermatitis** | | | | | | |
| --- | --- | --- | --- | --- | --- | --- |
| **Outcomes** | **Illustrative comparative risks* (95% CI)** | | **Relative effect (95% CI)** | **Participants (studies)** | **Quality of the evidence** | **Comments** |
|  | Assumed risk | Corresponding risk |  |  |  |  |
|  | **Placebo** | **JKIs** |  |  |  |  |
| **IGA Response_Li CY (JKIs vs. Placebo/Vehicle)** | **69 per 1000** | **196 per 1000** (155 to 246) | **RR 2.83**  (2.25 to 3.56) | 3525 (12 studies) | ⊕⊕⊕⊕ **high**^1,5,6,7^ | ^1^ Judged by ROB-1, "low risk"  ^5^ I^2^=0%  ^6^ N＞300 and 95% [2.25, 3.56]  ^7^ RR＞2,95% CI [2.25,3.56] |
| **IGA-Response_Tsai HR (Systemic JKIs vs. Placebo)** Follow-up: 4~16 weeks | **98 per 1000** | **265 per 1000** (190 to 371) | **RR 2.71**  (1.94 to 3.79) | 1534 (6 studies) | ⊕⊕⊕⊕ **high**^10,11^ | ^10^ Judged by ROB-2, "low risk" ^11^ I^2^=28% |
| **IGA-Response_Tsai HR (Topical JKIs vs. Vehicle)** Follow-up: 4~16 weeks | **59 per 1000** | **235 per 1000** (135 to 407) | **RR 4.01**  (2.31 to 6.95) | 883 (5 studies) | ⊕⊕⊕⊕ **high**^10,13,14^ | ^10^ Judged by ROB-2, "low risk"  ^13^ I^2^=0%  ^14^ RR=4.01, 95%CI [ 2.31,6.95] |
| **IGA-Response_Liu SQ (Abrocitinib vs. Placebo)** Follow-up: mean 12 weeks | **130 per 1000** | **393 per 1000** (294 to 523) | **RR 3.02**  (2.26 to 4.02) | 1825 (12 studies) | ⊕⊕⊕⊕ **high**^1,15,16^ | ^1^ Judged by ROB-1, "low risk"  ^15^ I^2^=51% ^16^ RR=3.02, 95% CI [2.26, 4.02] |
| **IGA-Response_Bikash (Abrocitinib vs. Placebo)** Follow-up: mean 12 weeks | **102 per 1000** | **358 per 1000** (283 to 454) | **RR 3.52**  (2.78 to 4.46) | 1843 (8 studies) | ⊕⊕⊕⊕ **high**^5,10,18^ | ^5^ I^2^=0%  ^10^ Judged by ROB-2, "low risk"  ^18^ RR=3.52, 95%CI [2.78,4.46] |
| **IGA-Response_Bikash (Abrocitinib vs. Placebo)-100mg** Follow-up: mean 12 weeks | **102 per 1000** | **299 per 1000** (214 to 420) | **RR 2.94**  (2.10 to 4.13) | 909 (4 studies) | ⊕⊕⊕⊕ **high**^5,10,19^ | ^5^ I^2^=0%  ^10^ Judged by ROB-2, "low risk"  ^19^ RR=2.94,95% CI [2.10,4.13] |
| **IGA-Response_Bikash (Abrocitinib vs. Placebo)-200mg** Follow-up: mean 12 weeks | **102 per 1000** | **426 per 1000** (305 to 591) | **RR 4.18**  (3.00 to 5.81) | 934 (4 studies) | ⊕⊕⊕⊕ **high**^5,10,20^ | ^5^ I^2^=0%  ^10^ Judged by ROB-2, "low risk"  ^20^ RR=4.18, 95% CI [3.00,5.81] |
| **IGA-Response_Hammad (Abrocitinib vs. Placebo)-100mg** Follow-up: 12~20 weeks | **97 per 1000** | **295 per 1000** (208 to 418) | **RR 3.03**  (2.14 to 4.30) | 924 (4 studies) | ⊕⊕⊕⊕ **high**^5,21,22^ | ^5^ I^2^=0%  ^21^ Judged by ROB-1, one was "high risk", one was "moderate risk", two were "low risk" ^22^ RR=3.03,95% CI [2.14,4.30] |
| **IGA-Response_Hammad (Abrocitinib vs. Placebo)-200mg** Follow-up: 12~20 weeks | **97 per 1000** | **432 per 1000** (307 to 607) | **RR 4.44**  (3.16 to 6.24) | 906 (4 studies) | ⊕⊕⊕⊕ **high**^21,23^ | ^21^ Judged by ROB-1, one was "high risk", one was "moderate risk", two were "low risk"  ^23^ RR=4.44,95% CI [3.16,6.24] |
| **IGA-Response_Wang B (Baricitinib vs. Placebo)** Follow-up: mean 16 weeks | **100 per 1000** | **194 per 1000** (155 to 242) | **RR 1.94**  (1.55 to 2.42) | 2595 (6 studies) | ⊕⊕⊕⊕ **high**^10,24^ | ^10^ Judged by ROB-2, "low risk"  ^24^ Dose-Response Gradient |
| **EASI75_Tsai HR (Systemic JKIs vs. Placebo)** Follow-up: 4~16 weeks | **113 per 1000** | **298 per 1000** (226 to 394) | **RR 2.64**  (2.00 to 3.49) | 2940 (9 studies) | ⊕⊕⊕⊕ **high**^10,25,26^ | ^10^ Judged by ROB-2, "low risk"  ^25^ I^2^=45%  ^26^ RR=2.64, 95% CI [2.00, 3.49] |
| **EASI75_Tsai HR (Topical JKIs vs. Vehicle)** Follow-up: mean 4 weeks | **76 per 1000** | **355 per 1000** (186 to 675) | **RR 4.69**  (2.46 to 8.92) | 558 (3 studies) | ⊕⊕⊕⊝ **moderate**^5,10,17^ | ^5^ I^2^=0%  ^10^ Judged by ROB-2, "low risk"  ^17^ Wide confidence interval |
| **EASI75_Bikash (Abrocitinib vs. Placebo)** Follow-up: mean 12 weeks | **180 per 1000** | **602 per 1000** (456 to 792) | **RR 3.35**  (2.54 to 4.41) | 1842 (8 studies) | ⊕⊕⊕⊕ **high**^10,30,31^ | ^10^ Judged by ROB-2, "low risk"  ^30^ I^2^=55% ^31^ RR=3.35, 95% CI [2.54,4.41] |
| **EASI75_Bikash (Abrocitinib vs. Placebo)-100mg** Follow-up: mean 12 weeks | **180 per 1000** | **492 per 1000** (357 to 681) | **RR 2.74**  (1.99 to 3.79) | 934 (4 studies) | ⊕⊕⊕⊕ **high**^10,32^ | ^10^ Judged by ROB-2, "low risk"  ^32^ I^2^=30% |
| **EASI75_Bikash (Abrocitinib vs. Placebo)-200mg** Follow-up: mean 12 weeks | **180 per 1000** | **726 per 1000** (458 to 1000) | **RR 4.04**  (2.55 to 6.42) | 908 (4 studies) | ⊕⊕⊕⊕ **high**^33,34^ | ^33^ I^2^=65% ^34^ RR=4.04,95% [2.55,6.42] |
| **EASI75_Wang B (Baricitinib vs. Placebo)** Follow-up: mean 16 weeks | **177 per 1000** | **319 per 1000** (271 to 374) | **RR 1.8**  (1.53 to 2.11) | 2595 (6 studies) | ⊕⊕⊕⊕ **high**^5,10,24^ | ^5^ I^2^=0%  ^10^ Judged by ROB-2, "low risk"  ^24^ Dose-Response Gradient |
| **PP-NRS_Liu SQ (Abrocitinib vs. Placebo)** Follow-up: mean 12 weeks | **212 per 1000** | **493 per 1000** (399 to 609) | **RR 2.32**  (1.88 to 2.87) | 1825 (5 studies) | ⊕⊕⊕⊕ **high**^1,24,35^ | ^1^ Judged by ROB-1, "low risk"  ^24^ Dose-Response Gradient  ^35^ I^2^=53% |
| **PP-NRS_Bikash (Abrocitinib vs. Placebo)** Follow-up: mean 12 weeks | **211 per 1000** | **535 per 1000** (411 to 695) | **RR 2.54**  (1.95 to 3.30) | 1789 (8 studies) | ⊕⊕⊕⊕ **high**^10,24,37^ | ^10^ Judged by ROB-2, "low risk"  ^24^ Dose-Response Gradient  ^37^ I^2^=58% |
| **PP-NRS_Bikash (Abrocitinib vs. Placebo)-100mg** Follow-up: mean 12 weeks | **211 per 1000** | **461 per 1000** (318 to 669) | **RR 2.19**  (1.51 to 3.18) | 901 (4 studies) | ⊕⊕⊕⊝ **moderate**^10,38^ | ^10^ Judged by ROB-2, "low risk"  ^38^ I^2^=54% |
| **PP-NRS_Bikash (Abrocitinib vs. Placebo)-200mg** Follow-up: mean 12 weeks | **211 per 1000** | **615 per 1000** (423 to 897) | **RR 2.92**  (2.01 to 4.26) | 888 (4 studies) | ⊕⊕⊕⊕ **high**^10,39,40^ | ^10^ Judged by ROB-2, "low risk"  ^39^ I^2^=58%  ^40^ RR=2.92,95% CI [2.01,4.26] |
| **PP-NRS_Hammad (Abrocitinib vs. Placebo)-100mg** Follow-up: 12~20 weeks | **212 per 1000** | **459 per 1000** (320 to 662) | **RR 2.17**  (1.51 to 3.13) | 913 (4 studies) | ⊕⊝⊝⊝ **very low**^21,41^ | ^21^ Judged by ROB-1, one was "high risk", one was "moderate risk", two were "low risk"  ^41^ I^2^=86% |
| **PP-NRS_Hammad (Abrocitinib vs. Placebo)-200mg** Follow-up: 12~20 weeks | **212 per 1000** | **550 per 1000** (284 to 1000) | **RR 2.60**  (1.34 to 5.05) | 898 (4 studies) | ⊕⊝⊝⊝ **very low**^17,21,42^ | ^17^ Wide confidence interval  ^21^ Judged by ROB-1, one was "high risk", one was "moderate risk", two were "low risk"  ^42^ I^2^=84% |
| *The basis for the **assumed risk** (e.g. the median control group risk across studies) is provided in footnotes. The **corresponding risk** (and its 95% confidence interval) is based on the assumed risk in the comparison group and the **relative effect** of the intervention (and its 95% CI).  **CI:** Confidence interval; **RR:** Risk ratio; **OR:** Odds ratio; | | | | | | |
| GRADE Working Group grades of evidence **High quality:** Further research is very unlikely to change our confidence in the estimate of effect.  **Moderate quality:** Further research is likely to have an important impact on our confidence in the estimate of effect and may change the estimate. **Low quality:** Further research is very likely to have an important impact on our confidence in the estimate of effect and is likely to change the estimate. **Very low quality:** We are very uncertain about the estimate. | | | | | | |
